# Novel transcriptome networks are associated with adaptation of capsicum fruit development to a light-blocking glasshouse film

**DOI:** 10.3389/fpls.2023.1280314

**Published:** 2023-11-06

**Authors:** Xin He, Celymar A. Solis, Sachin G. Chavan, Chelsea Maier, Yuanyuan Wang, Weiguang Liang, Norbert Klause, Oula Ghannoum, Christopher I. Cazzonelli, David T. Tissue, Zhong-Hua Chen

**Affiliations:** ^1^ National Vegetable Protected Cropping Centre, Hawkesbury Institute for the Environment, Western Sydney University, Penrith, NSW, Australia; ^2^ School of Science, Western Sydney University, Penrith, NSW, Australia; ^3^ Hubei Insect Resources Utilization and Sustainable Pest Management Key Laboratory, College of Plant Science and Technology, Huazhong Agricultural University, Wuhan, China; ^4^ Global Centre for Land Based Innovation, Western Sydney University, Richmond, NSW, Australia

**Keywords:** *Capsicum annuum* L., glasshouse covering material, low light, transcriptome, light receptors, fruit development, metabolic pathway

## Abstract

Light-blocking films (LBFs) can contribute to significant energy savings for protected cropping via altering light transmitting, such as UVA, photosynthetically active radiation, blue and red spectra affecting photosynthesis, and capsicum yield. Here, we investigated the effects of LBF on orange color capsicum (O06614, *Capsicum annuum* L.) fruit transcriptome at 35 (mature green) and 65 (mature ripe) days after pollination (DAP) relative to untreated control in a high-technology glasshouse. The results of targeted metabolites showed that LBF significantly promotes the percentage of lutein but decreased the percentage of zeaxanthin and neoxanthin only at 35 DAP. At 35 DAP, fruits were less impacted by LBF treatment (versus control) with a total of 1,192 differentially expressed genes (DEGs) compared with that at 65 DAP with 2,654 DEGs. Response to stress and response to light stimulus in biological process of Gene Ontology were found in 65-DAP fruits under LBF vs. control, and clustering analysis revealed a predominant role of light receptors and phytohormone signaling transduction as well as starch and sucrose metabolism in LBF adaptation. The light-signaling DEGs, UV light receptor *UVR8*, transcription factors *phytochrome-interacting factor 4* (*PIF4*), and an *E3 ubiquitin ligase* (*COP1*) were significantly downregulated at 65 DAP. Moreover, key DEGs in starch and sucrose metabolism (*SUS*, *SUC*, and *INV*), carotenoid synthesis (*PSY2* and *BCH1*), ascorbic acid biosynthesis (*VTC2*, *AAO*, and *GME*), abscisic acid (ABA) signaling (*NCED3*, *ABA2*, *AO4*, and *PYL2/4*), and phenylpropanoid biosynthesis (*PAL* and *DFR*) are important for the adaptation of 65-DAP fruits to LBF. Our results provide new candidate genes for improving quality traits of low-light adaptation of capsicum in protected cropping.

## 1 Introduction

Assuring a sustainable food supply is a big challenge for agriculture exposed to rising populations and extreme weather conditions under climate change (Gruda et al., 2019; Nicholson et al., 2021). Controlled environment glasshouses can improve sustainable and nutritious vegetable production (Gruda et al., 2019; He et al., 2021; Nicholson et al., 2021), but one of the major limitations is high energy use in protected cropping facilities, which may be reduced by using light-blocking dilm (LBF) (Cossu et al., 2014; Ezzaeri et al., 2018; Gao et al., 2019). In previous studies, LBF promoted energy use efficiency via reducing light transmission, such as significantly decreased daily light integral (DLI) and photosynthetically active radiation (PAR; 400–700 nm) (Chavan et al., 2020; Lin et al., 2022; Chavan et al., 2023). Changes of light quantity and quality inside the greenhouse influence fruit development, yield, and nutrient accumulation (Alsadon et al., 2016; Cossu et al., 2016; Ezzaeri et al., 2018; Hassanien et al., 2018; Ntinas et al., 2019; Chavan et al., 2020). Understanding how altered light conditions transmitted by protected cropping films impact molecular processes during fruit development can improve knowledge of the physiological processes underpinning crop productivity.

Capsicum (*Capsicum annuum* L.), also known as bell pepper, is one of the most economically important horticultural crops with high nutrition values, including carotenoids, ascorbic acid, and minerals (Carrara et al., 2001; Alagoz et al., 2018; Anwar et al., 2021). Capsicum fruit development strongly relies on light (Yamamoto et al., 2008; Hwang et al., 2020) while shade alters carbohydrate accumulation and the fruit set pattern, which delays ripening and reduces yield (Aloni et al., 1996; Chavan et al., 2020). The adaptation of fruits to light quality and shade conditions aligns with changes in physiology and metabolite levels (Díaz-Pérez, 2014; Ma and Li, 2019), including total titratable acids (Ilić et al., 2012; Chavan et al., 2020; Milenković et al., 2020; He et al., 2022; Chavan et al., 2023), ascorbate acid, and photosynthetic pigments (Alós et al., 2013; He et al., 2022). Metabolic changes can affect fruit color and taste, and there are genotypic differences in low-light–induced change of fruit quality traits (Do Rêgo et al., 2011; Díaz-Pérez, 2014; He et al., 2022). Our recent study showed that fruit quality traits such as color and ascorbic acid within the orange capsicum cultivar (O06614) were decreased by LBF without a yield penalty (He et al., 2022; Chavan et al., 2023). However, the underlying molecular mechanisms interrelated with these metabolic changes caused by the LBF remain unknown.

Interactions between photoreceptors and phytohormones play crucial roles in fruit set initiation, growth, and maturation. Photoreceptors [e.g., UV resistance locus 8 (UVR8), phytochromes (PHYs), phototropins (PHOTs), cryptochromes (CRYs), and zeitlupes (ZTLs)] and light signal regulators such as an E3 ubiquitin ligase CONSTITUTIVE PHOTOMORPHOGENIC 1 (COP1), and transcriptional factors [phytochrome-interacting factors (PIFs) and ELONGATED HYPOCOTYL 5 (HY5)] underpin transcriptomic changes in fruits development during altered light-growing conditions (Lunn et al., 2013; Fukushima et al., 2018; Shinozaki et al., 2018). Transcriptomics showed that phytohormones and sucrose metabolism interact with light-signaling function at different stages of fruit development (Watson et al., 2002; Osorio et al., 2012). PHYs and PIFs are involved in initial cell division and expansion of fruit set through genes that modulate phytohormone signaling genes for auxins (*Aux/IAA* and *ARF*), gibberellins (*GI*), ethylene (*EIN3* and *ACS*), and cytokinin (*CKI1*) signaling (Dobisova et al., 2017; Liu et al., 2018; Renau-Morata et al., 2020; Kuhn et al., 2021). As non-climacteric fruits, capsicum does not ripen after harvest, which depends more on the interaction of abscisic acid (ABA) and ethylene during the ripening process (Galpaz et al., 2008; Leng et al., 2009). Although ABA plays a crucial role in the developmental and environmental adaptation processes of plants (Chen et al., 2020), the molecular mechanisms of light receptor regulation of ABA are still not resolved.

Fruit ripening is a complex, genetically programmed, and environmentally regulated process. Low light limits the transport of photosynthates from leaves to fruit, which affects secondary metabolism during fruit ripening (Chavan et al., 2020). Invertase activities determine the accumulation of assimilates and the regulation of the sink metabolism of young fruit tissue. Reactivation of acid invertase and sucrose synthase (SUS) are responsible for the accumulation of hexoses during ripening (Vighi et al., 2019; Ji et al., 2020). Methylerythritol 4-phosphate/terpenoid and shikimate/phenylpropanoid pathways demonstrated that there are multiple levels of metabolic processes during fruit ripening (Wahyuni et al., 2013). Light-signaling transduction genes, such as *COP1*, *PIFs*, and *HY5*, participated in the regulation of key metabolic pathways (e.g., flavonoid or phenylpropanoid) and synthesis of anthocyanin, ascorbic acid, and carotenoids (Weatherwax et al., 1996; Xu et al., 2018; Xie et al., 2019; Zhang et al., 2021). However, signaling cascades of photoreceptors for fruit development could depend on the maturity stage (Simkin et al., 2020; Zhang et al., 2021). Furthermore, the interaction of light and hormones independently regulates multiple transcription factors (e.g., *MYBs*, *NACs*, *WRKY*s), creating a positive feedback regulatory circuit for the metabolism of carotenoids and flavonoid in fruit ripening during light adaption (Kadomura-Ishikawa et al., 2015; Wu et al., 2020; Yu et al., 2021).

In the present study, an orange capsicum (O06614, *Capsicum annuum* L.) was cultivated under the low light generated by LBF in the environmentally controlled greenhouse. We demonstrate how orange capsicum fruit development and key nutritional qualities of ripe fruit adapt to low light under LBF. The effect of LBF on molecular mechanisms regulating mature green (35 DAP) and ripe (65 DAP) stages of fruit development was investigated utilizing RNA-sequencing (RNA-seq) transcriptomics approach and carotenoid metabolites. The Gene Ontology (GO) of differentially expressed genes (DEGs) was assessed and network interactions interrelated to reveal candidate genes potentially regulating changes in physiological and metabolic processes. We hypothesize that genes and metabolites related to photoreceptors and phytohormones participate in orange fruit development in low-light adaptation under the LBF. This study represents a paradigm for exploring the differential expression of candidate genes and accumulation of nutrient components of an important horticultural crop, which provides a theoretical basis for selection of new crop varieties to better adapt to the reduced light environment in greenhouses with energy-saving films.

## 2 Materials and methods

### 2.1 Plant material and experimental treatment

**Figure 1:**
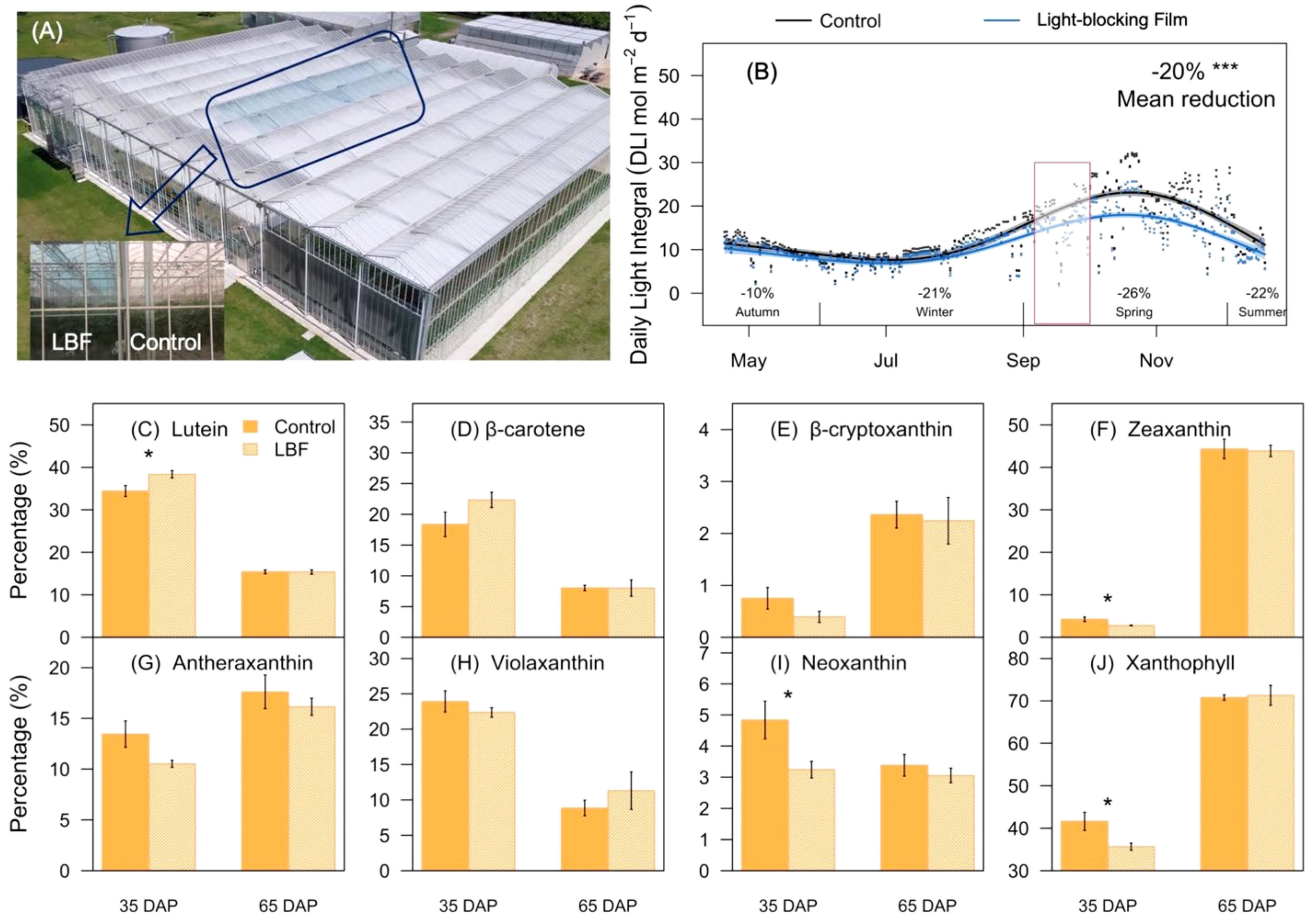
Schematic diagram, daily light integral (DLI), and representative carotenoids under light-blocking film (LBF) and control. **(A)** Schematic of experimental LBF coated and control chambers in a high-technology glasshouse. **(B)** DLI changes during cultivation of the capsicum crop (cv. O06614). The red box indicates the fruit sampling period in the production season. The percentage of each carotenoid component, including lutein **(C)**, β-carotene **(D)**, β-cryptoxanthin **(E)**, zeaxanthin **(F)**, antheraxanthin **(G)**, violaxanthin **(H)**, neoxanthin **(I)**, xanthophyll **(J)** was identified in 35 days after pollination (DAP) and 65 DAP of capsicum fruit under LBF and control. The significance levels were *, *** indicated P ≤ 0.05 and P ≤ 0.001.

The experiment was conducted in a high-technology glasshouse with an east–west orientation fitted with HD1AR diffuse glass (70% haze; roof and sidewall Glass covered 70% and 5%, respectively), located at the Hawkesbury Campus of Western Sydney University, Richmond, NSW, Australia. Two bays (105 m^2^ each) of the facility were coated with an LBF (ULR-80, Solar Gard, Saint-Gobain Performance Plastics, Sydney, Australia) on both roof and side walls as LBF treatment, and two bays were used as control as described by Chavan et al. (2020); Zhao et al. (2021), and He et al. (2022) (**Figure 1A**). The seed of Orange capsicum genotype (O06614) was sown into Rockwool and transplanted to Rockwool slabs cubes (Grodan, The Netherlands) on 19 April 2019, 42 days after sowing. The experiment lasted for 8 months with final harvest on 19 December 2019.

Flowers were labeled and recorded on the first day of blooming in the middle of July and the beginning of August 2019. Fruit samples were harvested 35 days after pollination (DAP; mature green) and 65 DAP (rip orange) in the morning (8:00~10:00; 20°C~25°C) at the end of September (140~150 days after planting) based on average fruit weight and color (He et al., 2022). According to the growth trend, the two developmental stages of fruit samples were collected from the 10th to 12th truss position (counting acropetally) and harvested with three biological replicates of each developmental stage under LBF and control. The area of each fruit (2 cm^2^) was randomly cut into pieces and then immediately frozen in liquid nitrogen and stored at −80°C until further analysis. The difference of light environment between the LBF and control during sampling was illustrated (**Figure 1B**). Similar cutting and pruning were used for vertical hydroponic cultivation under non-limiting water and nutrient (Electrical Conductivity (EC): 2.5~3.0 dS m^−1^, pH 5.0–5.5) conditions.

### 2.2 RNA extraction and quality control for RNA sequencing

A total of 12 samples were prepared for RNA-seq, including three replicates per treatment in each developmental stage. The samples of each replicate were ground to prepare RNA samples. An RNeasy Plant mini kit (Qiagen, Hilden, Germany) was used for RNA extraction to avoid sugar and phenolics interference. The quality of RNA samples was measured by the QIAxcel system (Qiagen, Germany). RNA samples were sequenced by Illumina paired-end sequencing (GENEWIZ Co., Ltd.) with the standard of Optical Density (OD) 260/280 ≥2.0 and RNA integrity number ≥8.0.

### 2.3 Transcriptome sequencing and enrichment analysis

Raw reads were used for preliminary analysis of the original image by Bcl2fastq (v2.17.1.14), and data were filtered by Cutadapt (version 1.9.1). The reference *Capsicum annuum* genome (Pepper cultivar Zunla 1 Ref_v1.0; https://www.ncbi.nlm.nih.gov/assembly/GCF_000710875.1) was selected to undergo the aligned analysis, and short-read alignment was performed using Hisat2 (v2.0.1) (Kim et al., 2015) with default parameters. Gene expression was calculated by transcripts per million (TPM) with the formula (Mortazavi et al., 2008):


$$\text{TPM}=\text{A}\times \frac{1}{\sum \left(\text{A}\right)}\times 10^{6}$$

$$Where\;A=\frac{\text{total reads mapped to gene}\times 10^{3}}{\text{gene length in bp}}$$

Differential GO against the genomic background was performed by Shinny GO (version 0.76.1; http://bioinformatics.sdstate.edu/go/) and returned three ontologies that describe the molecular function (MF), cellular component (CC), and biological process (BP) of the gene. The threshold for filtering was p-value ≤ 0.05. The GO terms (p-value ≤ 0.05) were used in the Plant Transcriptional Regulatory Map (Tian et al., 2020) to generate the diagrams in each term. The relative enrichment of the Kyoto Encyclopedia of Genes and Genomes (KEGG) was the primary public pathway database for Shinny GO. Pathway enrichment analysis was performed on the basis of KEGG pathway units and enrichment false discovery rate (FDR) ≤0.05. For each major stage, the Pearson correlation coefficient of all DEGs (in TPM values) with fluctuations of light receptors genes was calculated using the *cor* function in R, and the p-value was measured by the *cor.test* function in R (Zhang et al., 2020). The light-related genes were found in the same DEGs in both developmental stages responding to LBF. Only genes with significant Pearson correlation coefficient with p-value ≤0.01 were considered. Cytoscape (version 3.8.2; https://cytoscape.org/) was applied to screen the constructing light co-expression network selected from a correlation coefficient value ≥0.95 (p-value ≤ 0.001) (Shannon et al., 2003).

### 2.4 Carotenoid isolation in fruit samples by high-performance liquid chromatography

One hundred milligrams of 35-DAP and 65-DAP fruit pericarp (12 replicates of the orange genotype under LBF and control) were treated with liquid N_2_ and ground to a fine powder with a steel ball in 2-mL tubes by TissueLyser (Qiagen, Germany). Pigments were extracted under low-light conditions with 800 μL of extraction buffer (3/2, acetone/ethyl acetate), and 640 μL of H_2_O was added and centrifuged to separate the carotenoid-containing organic phase. The upper phase was transferred to a fresh tube and dried by SpeedyVac. The dry samples were resuspended in 750 μL of 100% tetrahydrofuran and 300 μL of 100% methanol. Then, 200 μL of 60% KOH (w/v; in 200 proof ethanol) was added to saponify carotenoid molecules. The saponified extractions were vortexed by adding 150 μL of 25% NaCl, 350 μL of 100% petroleum ether, and 300 μL of H_2_O, and the upper phase was resuspended in 300 μL of ethyl acetate. The samples were transferred to a vial and measured by reverse-phase high-performance liquid chromatography (HPLC) (Agilent 1200 Series, Santa Clara, USA) using GraceSmart-C30 (5 μm, 4.6 mm × 250 mm column; Alltech) column. The percentage of each component in total carotenoid content (%) after HPLC runs was calculated as previously described by Alagoz et al. (2020); Anwar et al. (2022), and He et al. (2022).

### 2.5 Gene set enrichment analysis

Gene set enrichment analysis (GSEA) was performed on the Run GSEA Pre-ranked using the GSEA software (version 4.3.1) (Mootha et al., 2003). The process has taken all gene expressions into consideration. It assumes that phenotypic differences are manifested by small but consistent changes in a set of genes. Normalized counts from DESeq2 were used as input data in the software. Gene sets were considered significantly enriched with FDR (*q*-values) ≤0.05 and nominal p-value ≤0.05 of the normalized enrichment scores.

### 2.6 Statistical analysis

All data were analyzed using the R 4.1.2 statistical computing environment (https://www.r-project.org/, 4.2.1; R Core Team, 2020). The construction of carotenoids was assessed for significant differences using Levene’s test from the *car* package with statistical significance considered if p-value ≤0.05. DEGs were calculated by *adgeR* and *DESeq2* packages to determine the criteria of fold change greater than 1.5 and a Q-value less than 0.05. Principal component analysis (PCA) and heatmaps were plotted by *prcomp* () function and *pheatmap* packages. Venn diagram was generated by *ggvenn* and *gridExtra* packages. Log_10_(TPM+1) of DEGs in fruit samples of the two developmental stages under LBF treatment was used for heatmaps with K-means cluster analysis, and data were plotted by *pheatmap*, *ggplot2*, *ggsignif*, and *grid*. Other R packages were also used, including *tidyverse* and *dplyr* for data manipulation, *cowplot* for generating various figures, and *doBy* for calculating means and standard errors.

## 3 Results

### 3.1 LBF significantly affects the composition of carotenoids in 35-DAP fruits

There were significant differences in the content and components of carotenoids during capsicum fruit development. The major constituents of carotenoid were lutein at 35 DAP, and LBF significantly promoted its proportion from 35% to 41% (P = 0.03, **Figures 1C–J**). Moreover, LBF significantly reduced the percentage of zeaxanthin (29.4%, P = 0.02), neoxanthin (31.9%, P = 0.01), and xanthophyll (31.0%, P = 0.02) at 35 DAP. During fruit ripening, zeaxanthin increased to become the dominant component of carotenoids reaching nearly 50%. However, LBF did not significantly affect the content and components of carotenoids at 65 DAP compared with those in the control. Overall, carotenoids accompanied by typical color changes during fruit development and LBF only influenced the components of carotenoid in 35-DAP fruit.

### 3.2 Global RNAseq analysis of LBF effects during capsicum fruit development

Transcriptome profiles were generated by RNA-seq and analysis at two developmental stages (35 DAP and 65 DAP) to assess the potential effects of LBF on capsicum fruits. In total, 12 libraries were constructed and analyzed after removing low-quality reads. The average reads per library were 45,532,101 with more than 94% of the total clean reads with a Phred-like quality score at Q30 level (**Supplementary Tables 1**, **2**). More than 92% of the total reads of all samples were successfully aligned with the reference map of the *Capsicum annuum* genome (Pepper Zunla 1 Ref_v1.0), and more than 87% were successfully uniquely mapped with an average Guanine-cytosine (GC) content of 44.0%. Overall, 23,332 genes were mapped and identified with TPM >0 in at least one of the 12 samples. The gene expression levels among biologically replicates were highly consistent.

**Figure 2:**
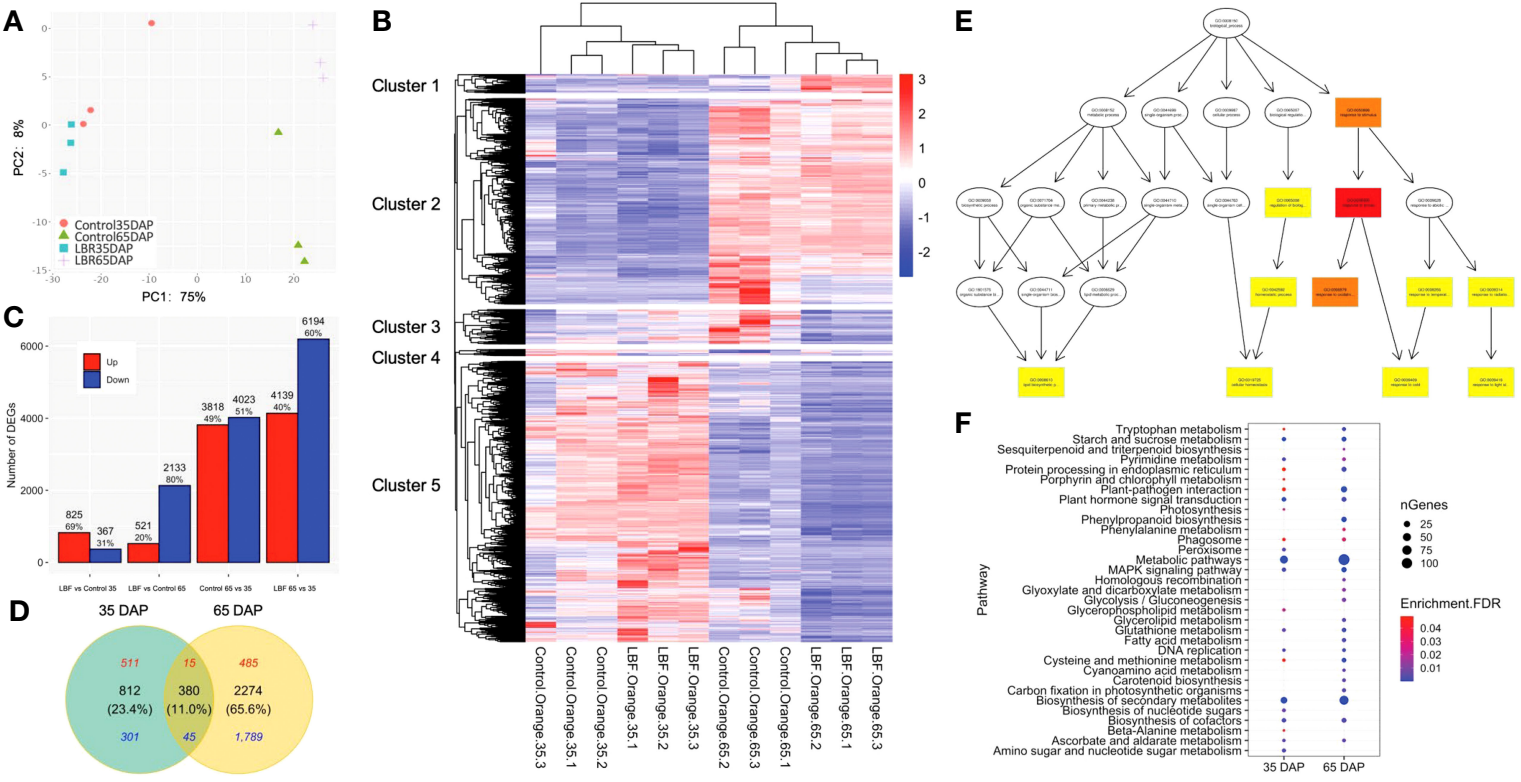
Overview of the differentially expressed genes (DEGs) in two developmental stages of capsicum under light-blocking film (LBF). **(A)** The numbers and percentage of up- and downregulated DEGs, which are selected on the basis of fold change >1.5 and FDR<0.05. **(B)** Venn diagrams of 35 DAP and 65 DAP of fruit under LBF vs. control and **(C)** principal component analysis (PCA) of 12 RNA-sequencing samples (n = 3 biological replicates for each treatment per stage). **(D)** Heatmap of DEGs [log_10_(TPM+1)] with K-means cluster analysis. Directed acyclic graph of biological progress **(E)** in 65 DAP under LBF versus control. The GO term with a deeper color represents more significant enrichment. Bubble charts of significantly enriched KEGG **(F)** in 35 DAP and 65 DAP under LBF versus control.

PCA indicated the separation between 35 DAP and 65 DAP, as well as between LBF treatment and control (**Figure 2A**). The heatmap results revealed that 12 samples were separated into five main clusters corresponding to the two developmental stages based on gene expression patterns (**Figure 2B**). For instance, DEGs in clusters 4 and 5 were highly expressed in 35-DAP fruits, whereas DEGs in clusters 1 and 2 were highly expressed in 65 DAP. DEGs in clusters 2 and 5 oppositely regulated during fruit ripening (**Supplementary Figure 1**). These results suggested that low light generated by LBF plays important roles in regulating DEGs at different fruit developmental stages.

More DEGs were found to be downregulated (5.8 folds) in 65-DAP fruit under LBF vs. control compared with those in 35-DAP fruit. A total of 3,846 DEGs were identified from the fruit samples of 35 DAP and 65 DAP under LBF treatment in comparison with the control, and 65 DAP had 69.0% more DEGs than those in 35 DAP (**Figure 2C**, **Supplementary Tables 3**-**5**). There were 521 upregulated and 2,133 downregulated DEGs in 65 DAP, but the corresponding numbers were 825 and 367 in 35 DAP, respectively. The Venn diagram showed that 380 shared DEGs (11.0%) included only 60 DEGs (15 upregulated and 45 downregulated DEGs) with same trends among the two stages, and the rest of the 320 DEGs were oppositely regulated between 35 DAP and 65 DAP under LBF vs. control (**Figure 2D**, **Supplementary Table 6**). Furthermore, 812 DEGs and 2,274 DEGs were typically in 35 DAP and 65 DAP response to LBF, respectively.

GO enrichment analysis divided DEGs of 35 DAP and 65 DAP under LBF treatment into eight and 61 GO terms, respectively (**Supplementary Table 7**). Eight GO terms in MF, including DNA-binding transcription factor activity (GO:0003700) and SUS activity (GO:0016157), were significantly enriched in 35 DAP under LBF vs. control. For increased GO terms found in 65-DAP fruits under LBF vs. control, cellular response to stress (GO:0033554), abiotic stimulus (GO:0009628), reactive oxygen species (ROS; GO:0000302), and response to light stimulus (GO:0009416) in BP revealed a predominant role of LBF adaptation (**Figure 2E**). Furthermore, ABA-activated signaling pathway (GO:0009738) in BP and ABA binding (GO:00104027) in MF were significantly enriched in 65-DAP fruits under LBF vs. control (**Supplementary Table 7**). All DEGs were assigned to 22 and 26 KEGG pathways in 35 DAP and 65 DAP, respectively, under LBF vs. control (**Supplementary Table 8**). DEGs in metabolic pathways accounted for the largest proportion, followed by biosynthesis of secondary metabolites (**Figure 2F**). Starch and sucrose metabolism, plant hormone signal transduction, DNA replication, and Mitogen-activated protein kinase (MAPK) signaling pathway were significantly enriched in the 35-DAP fruit after LBF treatment. With exception of these, more pathways were found in 65-DAP fruit under LBF treatment, such as plant-pathogen interaction, phenylpropanoid biosynthesis, and cysteine and methionine metabolism.

Differences in ripening process were examined in LBF and control under 65 DAP vs. 35 DAP. A total of 7,841 and 10,334 DEGs were observed in control and LBF treatment, respectively (**Figure 2A**, **Supplementary Tables 9**, **10**). A total of 47.9% of the DEGs (5,889) were the same under 65 DAP vs. 35 DAP in both control and LBF, with 2,378 upregulated and 3,311 downregulated during fruit ripening (**Supplementary Figure 2A**). A total of 1,952 DEGs and 4,444 DEGs were typically ripening-related in control and LBF, respectively. The largest proportion of those DEGs was enriched in metabolic pathways and biosynthesis of secondary metabolites (**Supplementary Table 8**). The enrichment of DEGs in plant hormone signal transduction as well as starch and sucrose metabolism was found in the top 10 KEGG pathways in 65 DAP vs. 35 DAP under LBF compared with amino sugar and nucleotide sugar metabolism and MAPK signaling pathway in that under control (**Supplementary Table 8**). DEGs enriched in RNA degradation, proteasome, protein export, pyrimidine and histidine metabolism, fatty acid elongation, arachidonic acid metabolism, stilbenoid, diarylheptanoid, and gingerol biosynthesis were typically found in fruit-ripening process under LBF (**Supplementary Figure 2B**).

### 3.3 Different light receptors and their signaling regulators regulate fruit development under LBF treatment

**Figure 3:**
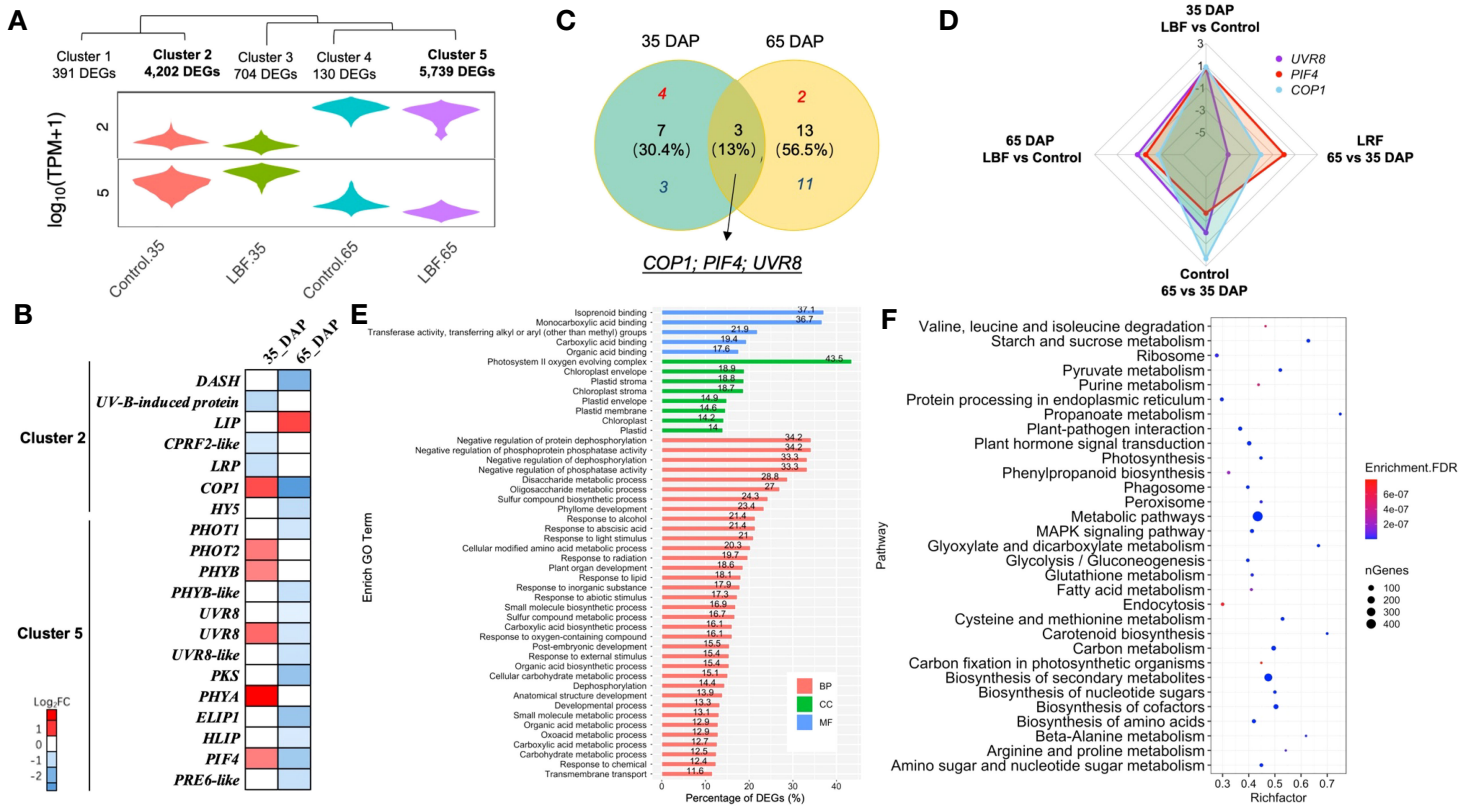
DEGs encoding light receptors and signal transduction in capsicum fruits adapted to light-blocking film (LBF). **(A)** Violin plot shows the relative cluster of light receptors. **(B)** Heatmap of light receptors and related signaling DEGs in each sample and both stages under LBF vs. control. **(C)** Venn diagram of the DEGs in both 35 DAP and 65 DAP of capsicum under LBF vs. control. **(D)** Radar chart of *UVR8*, *PIF4*, and *COP1* expressed in four comparisons. Bar **(E)** and bubble **(F)** charts illustrate the enrichment GO (FDR< 0.001) and KEGG (FDR< 10^−6^) of DEGs in clusters 2 and 5. *DASH*, *Drosophila*, *Arabidopsis*, *Synchesis*, *Human-type cryptochromes*; *LIP*, *light-induced protein*; *CPRF2-like*, *light-inducible protein CPRF2–like*; *LRP*, *light-regulated protein*; *COP1*, *E3 ubiquitin-protein ligase*; *HY5*, *ELONGATED HYPOCOTTYL 5*; *PHOT1/2*, *phototropin-1/2*; *PHYB/B-like*, *phytochrome B/B–like*; *UVR8/8-like*, *ultraviolet-B receptor 8/8–like*; *PKS*, *phytochrome kinase substrate* 1; *PHYA*, *light-sensor Protein kinase–like*; *ELIP1*, *early-light–induced protein 1*; *HLIP*, *high-light–induced protein*; *PIF4*, *PHYTOCHROME-INTERACTING FACTOR 4*; *PRE6-like*, *transcription factor PRE6–like*.

Light-related DEGs were in clusters 2 and 5 (**Figures 3A, B**). *UVR8*, *COP1*, and *PIF4* were found in both 35-DAP and 65-DAP fruits responding to LBF treatment, but they were upregulated in 35 DAP and downregulated in 65 DAP under LBF vs. control (**Figures 3C, D**). More light-related DEGs were investigated in 65 DAP with downregulation response to LBF (**Figures 3B, C**). In 35 DAP, *phototropin 2* (*PHOT2*), *phytochrome A* and *B* (*PHYA* and *PHYB*), *ultraviolet-B receptor* (*UVR8*), *Phytochrome-Interacting Factor 4* (*PIF4*), and E3 ubiquitin-protein ligase *Constitutive Photomorphogenic 1* (*COP1*) were upregulated [log_2_ fold change (log_2_FC): 0.63~1.3] after LBF treatment (**Figure 3B**, **Supplementary Table 11**); however, except *light-induced protein* (*LIP*; log_2_FC: 0.92), all the light-signaling transduction DEGs were downregulated (log_2_FC: −2.71~−0.59) in 65 DAP under LBF treatment. The DEGs in cluster 2 were downregulated in both 35 DAP and 65 DAP, whereas DEGs in cluster 5 were upregulated in 35 DAP and downregulated in 65 DAP. Those DEGs in clusters 2 and 5 were enriched in the response to light stimulus (GO:0009416), ABA-activated signaling pathway (GO:0009738), carbohydrate metabolic process (GO:0005975) in BP, ABA binding (GO:0010427) in MF, and plastid (GO:0009536) in CC (**Figure 3E**). Metabolic pathways, biosynthesis of secondary metabolites, biosynthesis of cofactors, plant hormone signal transduction, and starch and sucrose metabolism were the top five enriched KEGG pathways (**Figure 3F**).

**Figure 4:**
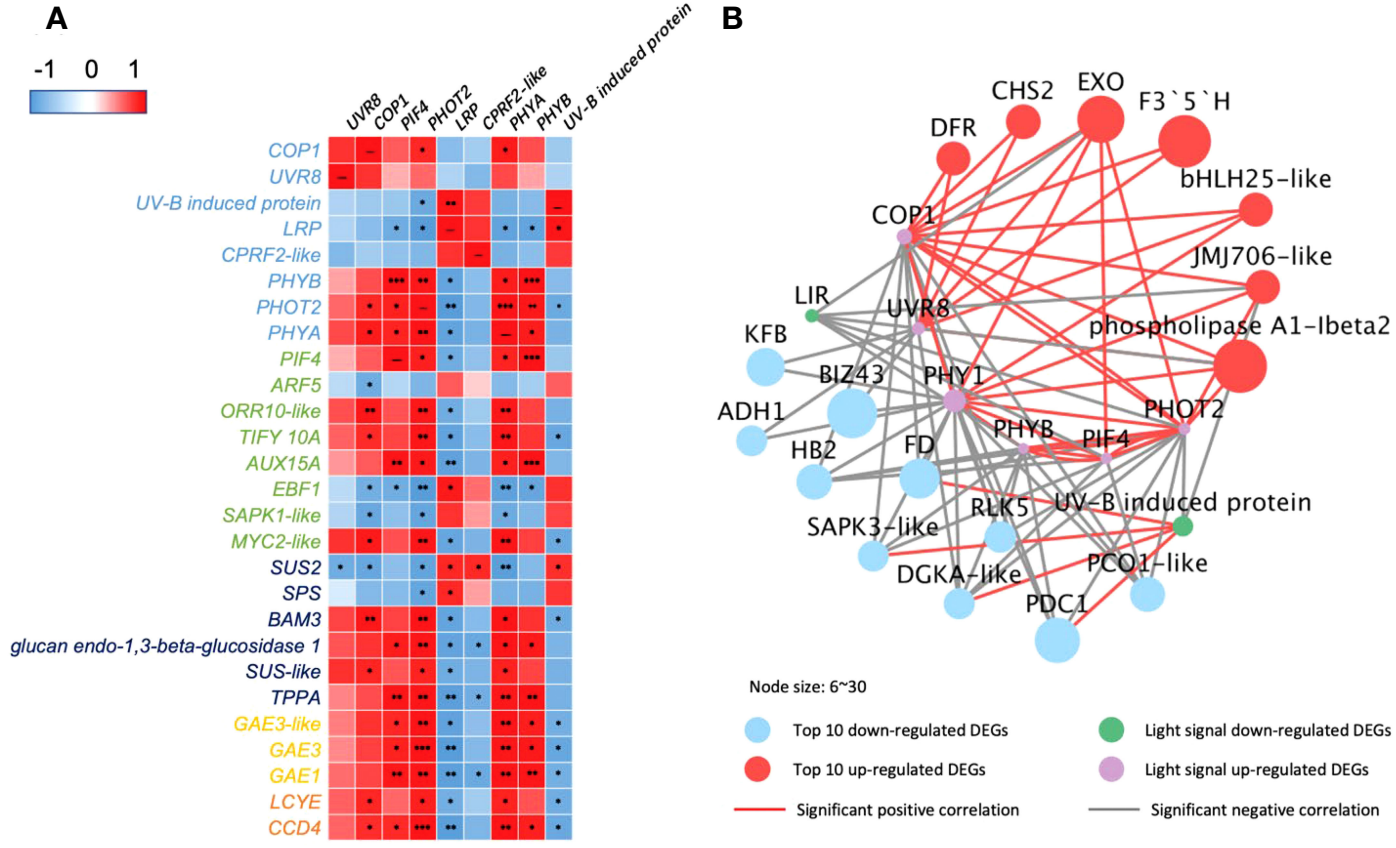
Correlations of DEGs encoding light receptors and their transduction factors in 35-DAP capsicum fruits. **(A)** Heatmap of DEGs in light-signaling and plant hormone transduction, starch and sucrose metabolism, ascorbate and aldarate metabolism, and carotenoid biosynthesis pathway. **(B)** Networks among light-signaling and top 10 up- and downregulated DEGs. The node size is set according to the 10 times log_2_FC value under LBF vs. control. Light receptors and signaling factors: *UVR8*, *ultraviolet-B receptor*; *COP1*, *E3 ubiquitin-protein ligase*; *LRP*, *light-regulated protein*; *CPRF2-like*, *light-inducible protein*; *PHYB*, *phytochrome B*; *PHOT2*, *phototropin-2*; *PHYA*, *light-sensor Protein kinase–like*. Phytohormone pathway (green color): *PIF4*, *PHYTOCHROME-INTERACTING FACTOR 4*; *ARF5*, *auxin response factor 5*; *ORR10-like*, *two-component response regulator*; *EBF1*, *EIN3-binding F-box protein 1–like*; *SAPK1-like*, *serine/threonine-protein kinase*; *TIFY 10A*, *protein TIFY 10A–like*; *AUX15A*, *auxin-induced protein 15A–like*. Starch and sucrose metabolism (dark blue color): *SUS2*, *sucrose synthase 2*; *SPS*, *sucrose-phosphate synthase*; *BAM3*, *beta-amylase 3*; *SUS-like*, *sucrose synthase–like*; *TPPA*, *trehalose-phosphate phosphatase A–like*. Ascorbate and aldarate metabolism (yellow color): *GAE1/3*, *UDP-glucuronate 4-epimerase 1/3*. Carotenoid biosynthesis (orange color): *LCYE*, *lycopene epsilon cyclase*; *CCD4*, *carotenoid cleavage dioxygenase* 4. Top downregulated DEGs: *KFB*, *kelch repeat-containing protein*; *BIZ43*, *basic leucine zipper 43*; *FD*, *ferredoxin*; *RLK5*, *receptor-like protein kinase 5*; *PCO1-like*, *plant cysteine oxidase 1–like*; *ADH1*, *alcohol dehydrogenase 1*; *DGKA-like*, *diacylglycerol kinase A–like*; *PDC1*, *pyruvate decarboxylase 1*. Top upregulated DEGs: *EXO*, *protein EXORDIUM–like*; *DFR*, *dihydroflavonol-4-reductase*; *JMJ706-like*, *lysine-specific demethylase*; *CHS2*, *chalcone synthase 2*; *F3′5′H*, *flavonoid 3’*,*5’-hydroxylase*. The significance levels were *, **, *** indicated P ≤ 0.05, P ≤ 0.01, and P ≤ 0.001.

The correlation analysis (**Figure 4A**) indicated that *PHOT2* and *PHYA* play an important role in 35-DAP fruit development correlated to plant hormone transduction, starch and sucrose metabolism, ascorbate and aldarate metabolism, and carotenoid metabolism. The upregulated *COP1* and *UVR8* had positive correlations with transcription factors *bHLH25-like* and *PIF4*, as well as significantly expressed with *dihydro flavonol-4-reductase* (*DFR*), *chalcone synthase 2* (*CHS2*), and *flavonoid 3’*,*5’-hydroxylase* (*F3′5′H*; **Figure 4B**). Lipid metabolism [such as *phospholipase A1-lbeta 2* and *diacylglycerol kinase A–like* (*DGKA-like*)], polyphenols [*kelch repeat-containing protein* (*KFB*)], and chromoplast redox metabolism [*ferredoxin* (*FD*)] were also significantly correlated to light-signaling genes in 35-DAP fruit LBF adaptation (**Figure 4**).

**Figure 5:**
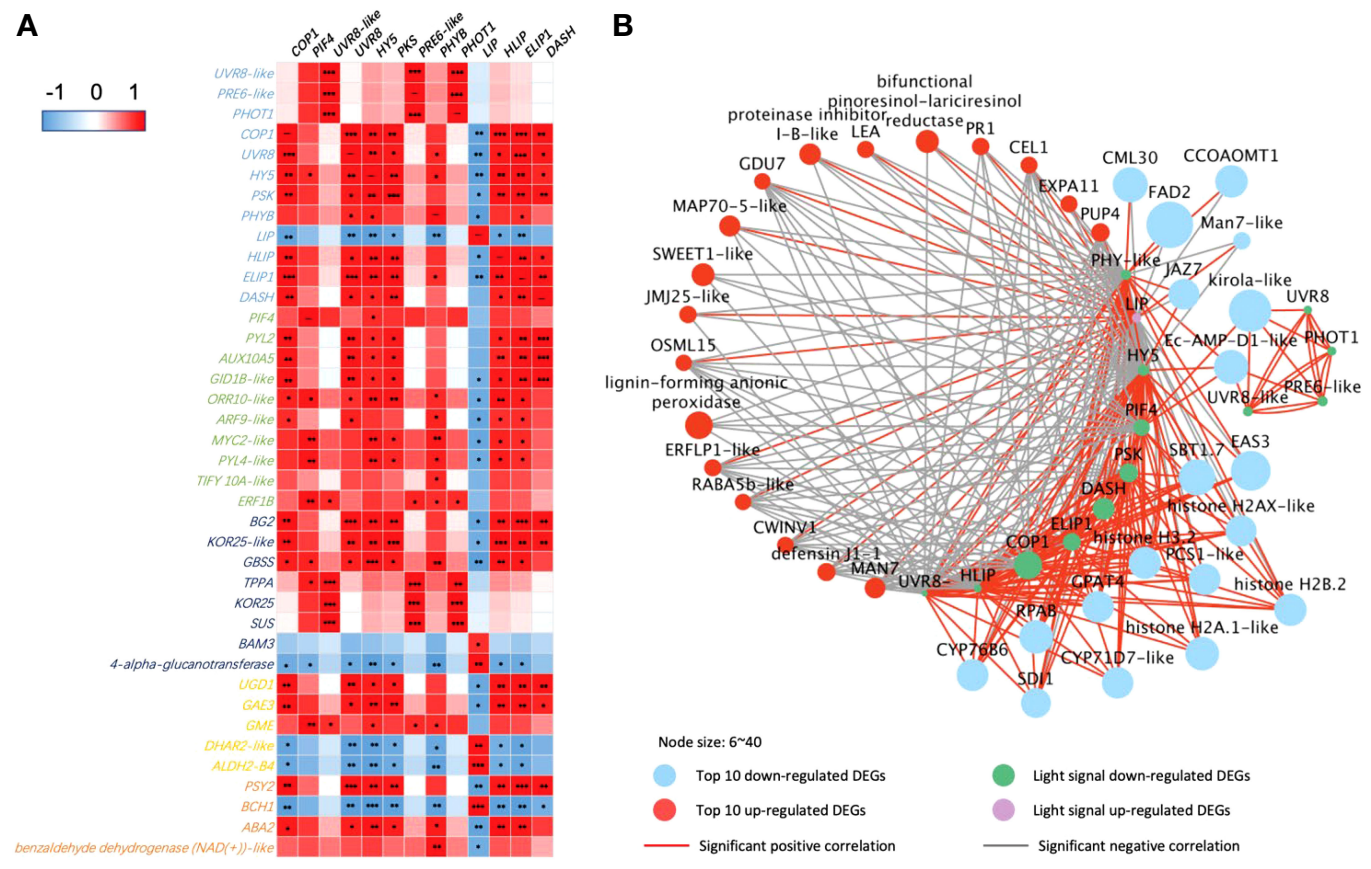
Correlations of DEGs encoding light receptors and their transduction factors in 65-DAP capsicum fruits. **(A)** Heatmap between DEGs of light-signaling and plant hormone transduction, starch and sucrose metabolism, ascorbate and aldarate metabolism, and carotenoid biosynthesis pathway. **(B)** Networks among light-signaling and top 20 up- and downregulated DEGs. The node size is set according to the log_2_FC under LBF vs. control. Light-signaling DEGs: *COP1*, *E3 ubiquitin-protein ligase Constitutive Photomorphogenic 1*; *DASH*, *Drosophila*, *Arabidopsis*, *Synechocystis*, *Human-type cryptochromes*; *ELIP1*, *early-light–induced protein 1*; *PSK*, *protein phytochrome kinase substrate 1*; *UVR8*, *ultraviolet-B receptor*; *HLIP*, *high-light–induced protein*; *HY5*, *ELONGATED HYPOCOTYL5*; *PHYB-like*, *phytochrome B–like*; *PHOT1*, *phototropin-1*; *LIP*, *light-induced protein*. Phytohormone pathway (green color): *PIF4*, *PHYTOCHROME-INTERACTING FACTOR 4*; *PYL2/PYL4-like*, *abscisic acid receptor*; *GID1B-like*, *gibberellin receptor*; *ORR10-like*, *two-component response regulator*; *ARF9-like*, *auxin response factor 9–like*; *ERF1B*, *ethylene-responsive transcription factor 1B–like*; *AUX15A*, *auxin-induced protein 15A–like*. Starch and sucrose metabolism (dark blue color): *BG2*, *glucan endo-1*,*3-beta-glucosidase 2*; *KOR25-like*, *endoglucanase 25–like*; *GBSS*, *granule-bound starch synthase 2*; *TPPA*, *trehalose-phosphate phosphatase A–like*; *SUS*, *sucrose synthase*; *BAM3*, *beta-amylase 3*. Ascorbate and aldarate metabolism (yellow color): *UGD1*, *UDP-glucose 6-dehydrogenase 1–like*; *GAE3*, *UDP-glucuronate 4-epimerase 3*; *GME*, *GDP-mannose 3*,*5-epimerase 1*; *DHAR2-like*, *glutathione S-transferase*; *ALDH2-B4*, *aldehyde dehydrogenase family 2 member B4*. Carotenoid biosynthesis (orange color): *PSY2*, *phytoene synthase 2*; *ABA2*, *xanthoxin dehydrogenase–like*; *BCH1*, *beta-carotene hydroxylase 1*. Top upregulated DEGs: *LEA*, *late embryogenesis abundant protein*; *Man7-like*, *mannan endo-1*,*4-beta-mannosidase 7–like*; *EXPA11*, *expansin-A11–like*; *JMJ25-like*, *lysine-specific demethylase*; *CWINV1*, *beta-fructofuranosidase*, *insoluble isoenzyme 1–like*; *MAP70-5-like*, *microtubule-associated protein 70-5–like*; *CEL1*, *endoglucanase 18–like*; *PUP4*, *probable purine permease 4*; *RABA5b-like*, *ras-related protein*; *GDU7*, *protein Glutamine Dumper 6–like*; *ERFLP1-like*, *ethylene-responsive proteinase inhibitor 1–like*; *OSML15*, *osmotin-like protein*; *PR1*, *pathogenesis-related protein 1A–like*. Top downregulated DEGs: *AP*, *aspartic proteinase*; *SBT1.7*, *subtilisin-like protease*; *GPAT4*, *glycerol-3-phosphate 2-O-acyltransferase 4*; *SDI1*, *protein Sulfur Deficiency–Induced 1*; *CYP71D7-like*, *cytochrome P450 71D7–like*; *EAS3*, *5-epiaristolochene synthase–like*; *CYP76B6*, *geraniol 8-hydroxylase–like*; *RPAB*, *replication protein A 70-kDa DNA-binding subunit B*; *FAD2*, *delta(12)-acyl-lipid-desaturase–like*; *JAZ7*, *jasmonate-zim-domin protein 7*; *CML30*, *calcium-binding protein*; *CCOAOMT1*, *caffeoyl-CoA O-methyltransferase–like*. The significance levels were *, **, *** indicated P ≤ 0.05, P ≤ 0.01, and P ≤ 0.001.

There are more light receptors significantly expressed with the key DEGs in secondary metabolic pathways in 65-DAP fruit under LBF treatment (**Figure 5**). *PHOT1*, *UVR8-like*, and transcription factor *PRE6–like* had a positive correlation with starch and sucrose metabolism such as *TPPA*, *endoglucanase 25–like* (*KOR25*), and *SUS* and downregulated in defensin such as *kirola-like* (**Figures 5A,B**). Another light network was constructed by *DASH*, *PHYB*, *PSK*, *UVR8*, *LIP*, *high-light–induced protein* (*HLIP*), and *early-light–induced protein* (*ELIP*), which were significantly expressed with plant hormone transduction, starch and sucrose metabolism, ascorbate and aldarate metabolism, and carotenoids metabolism (**Figure 5A**). Except for the upregulated *LIP*, *ELIP*, *DASH*, *PKS*, and *PHYB* significantly downregulated (log_2_FC = 1.1~2.1, p-value< 10^−5^), in 65 DAP under LBF treatment (**Supplementary Table 11**). *COP1*, *HY5*, and *PIF4* could mediate the light receptors downregulated the fruit-ripening process under low carbon availability (*TPPA* and *SUS*, *SWEET1-like*), fruit growth response to low light [such as *aspartic proteinase* (*AP*), *late embryogenesis abundant* (*LEA*), *jasmonate-zim-domain 7* (*JAZ7*), *expansin-A11–like* (*EXPA11*), and *cytochrome P450 71D7–like* (*CYP71D7-like*)], loose ripening-triggered cell wall [such as *mannan endo-1*,*4-beta-mannosidase 7–like* (*MAN7-like*); *beta-fructofuranosidase*, *insoluble isoenzyme 1–like* (*CWINV1*); and *endoglucanase 18–like* (*CEL1*)], lipid synthesis [*delta*(*12*)*-acyl-lipid-desaturase–like* (*FAD2*)], and mineral uptake [*Sulfur Deficiency–Induced* (*SDI2*) and *calcium-binding protein* (*CML30*)]. Moreover, defense [*MYC2-like*, *defensin J1-1*, and *pathogenesis-related protein 1A–like* (*PR1*)], ascorbate and aldarate metabolism [*GDP-mannose 3*,*5-epimerase 1* (*GME*) and *UDP-glucuronate 4-epimerase 3*], demethylase (*histones H2A*/*H3.2* and *lysine-specific demethylase JMJ25–like*), and phytohormone transduction [xanthoxin dehydrogenase*–*like (ABA2), *abscisic acid receptor PYL2/4*, and *ethylene-responsive proteinase inhibitor 1–like* (*ERFLP-like*)] were significantly correlated to upregulated LIP in 65 DAP under LBF treatment.

### 3.4 Phytohormone signaling pathway, phenylpropanoid biosynthesis, and ascorbate and aldarate metabolism in 65-DAP fruits were significantly downregulated by LBF

**Figure 6:**
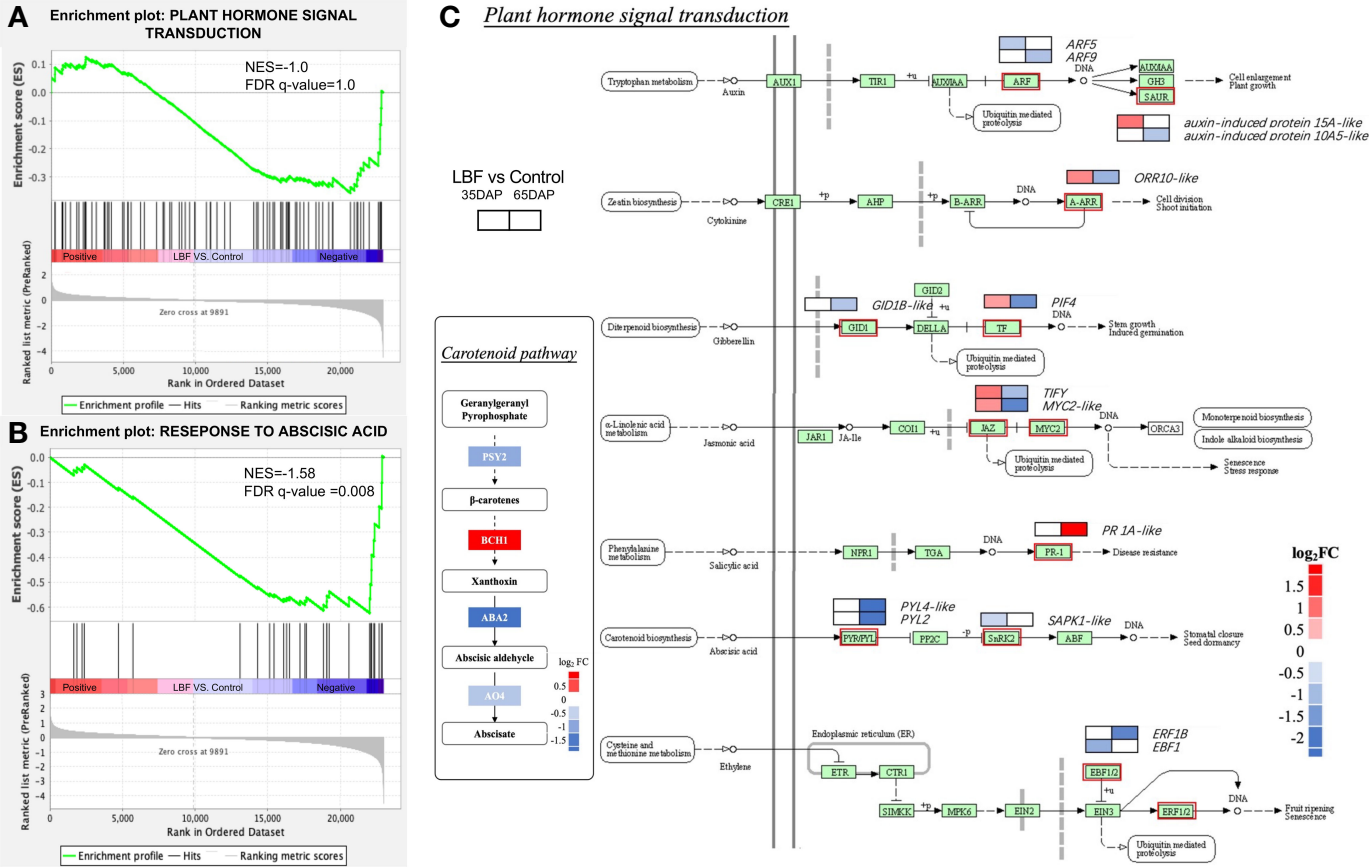
Effects of light-blocking film (LBF) on DEGs encoding plant hormone signal transduction in capsicum fruits. Gene set enrichment analysis (GSEA) results of plant hormone signal transduction (KEGG, **A**) and response to abscisic acid (GO, **B**). **(C)** Diagram of plant hormone signal transduction and carotenoid pathway in 65 DAP connected to ABA synthesis. The two blocks in the diagram indicate the log_2_FC in 35 DAP and 65 DAP under LBF vs. control. *PIF4*, *PHYTOCHROME-INTERACTING FACTOR 4*; *PYL2/PYL4-like*, *abscisic acid receptor*; *GID1B-like*, *gibberellin receptor*; *ORR10-like*, *two-component response regulator*; *ARF5/9-like*, *auxin response factor 5/9–like*; *ERF*, *ethylene-responsive transcription factor*; *PR 1A-like*, *pathogenesis-related protein 1A–like*; *PSY2*, *phytoene synthase 2*; *ABA2*, *xanthoxin dehydrogenase–like*; *BCH1*, *beta-carotene hydroxylase 1*; *AO4*, *aldehyde oxidase 4*.

We also found that 85 DEGs of the phytohormone signaling pathways were downregulated in 65-DAP fruit under LBF vs. control (**Figure 6**). The GSEA results represented normalized enrichment score (NES) of the phytohormone signaling pathway of −1.0 (p-value = 0.04, FDR = 1.0). A total of 42.4% genes in phytohormone signaling pathway were downregulated, including ABA receptor *PYL2*, *PYL4-like*, leading to the core enrichment, and the ABA signaling pathway significantly downregulated (NES = −1.58, FDR = 0.008), which was also found together with light signals relative DEGs in same clusters (**Figure 5A**, **Supplementary Table 12**). Diagram of plant hormone signal transduction showed that ABA biosynthesis DEGs are significantly downregulated, including *CYP707A1*, *ABA-deficient* (*ABA2*) and *AO4*, as well as ABA receptor genes *PYL2*, *PYL4-like*, and *SAPK1-like* (**Supplementary Tables 5**, **12**). Apart from that, *ethylene-responsive transcription factor 1B–like*, *transcription factor MYC2-like*, *PIF4*, and *TIFY* in ethylene and jasmonic acid pathways were significantly downregulated (log_2_FC = −1.78~1.58). *Auxin response factor* 5/9, *auxin-induced protein 10A5* (log_2_FC = −2.58~−0.82), and *two-component response regulator ORR10–like* (log_2_FC = −1.12) in cytokinin and *gibberellin receptor GID1B–like* (log_2_FC = −0.82) were also significantly downregulated.

Many antioxidants such as phenylpropanoid and ascorbates can spontaneously react with ROS to reduce the oxidative stress (**Supplementary Figures 3**, **4**). In phenylpropanoid biosynthesis, transcripts from a total of 45 genes were significant differentially expressed among fruit samples. Twenty-two genes, including *phenylalanine ammonia-lyase* (*PAL*), *cinnamic acid 4-hydroxylase* (*C4H*), *caffeic acid 3-Omethyltransferase* (*COMT*), *4-coumarate-CoAligase1* (*CHS*), *lignin-forming anionic peroxidase*, *cinnamoyl-CoA reductase 1–like* (*CCR1*), *peroxidase 42* (*POD42*), *beta-glucosidase 18–like* (*BGL18-like*), and *caffeoyl-CoA O-methyltransferase–like* (*CCOAOMT1*), were downregulated (log_2_FC = −3.16~-0.89) in the 65-DAP fruit in the decreased light under LBF (**Supplementary Table 12**). Those are the core genes that contributed to the downregulation of phenylpropanoid biosynthesis of 65-DAP fruits in LBF adaptation (NES = −1.66, FDR = 0.05). The downregulated *UDP-glucuronate 4-epimerase 3*, *UDP-glucose 6-dehydrogenase 1–like* (*UGD1*), and *GDP-mannose 3*,*5-epimerase 1* (*GME*; log_2_FC = −1.36~−1.17) were the core enrichment on ascorbate and aldarate metabolism (NES = −1.24, FDR = 0.97; **Supplementary Figure 3**) in 65-DAP fruit.

## 4 Discussion

### 4.1 Photoreceptors and light-signaling components respond differently to LBF treatment during fruit development

**Figure 7:**
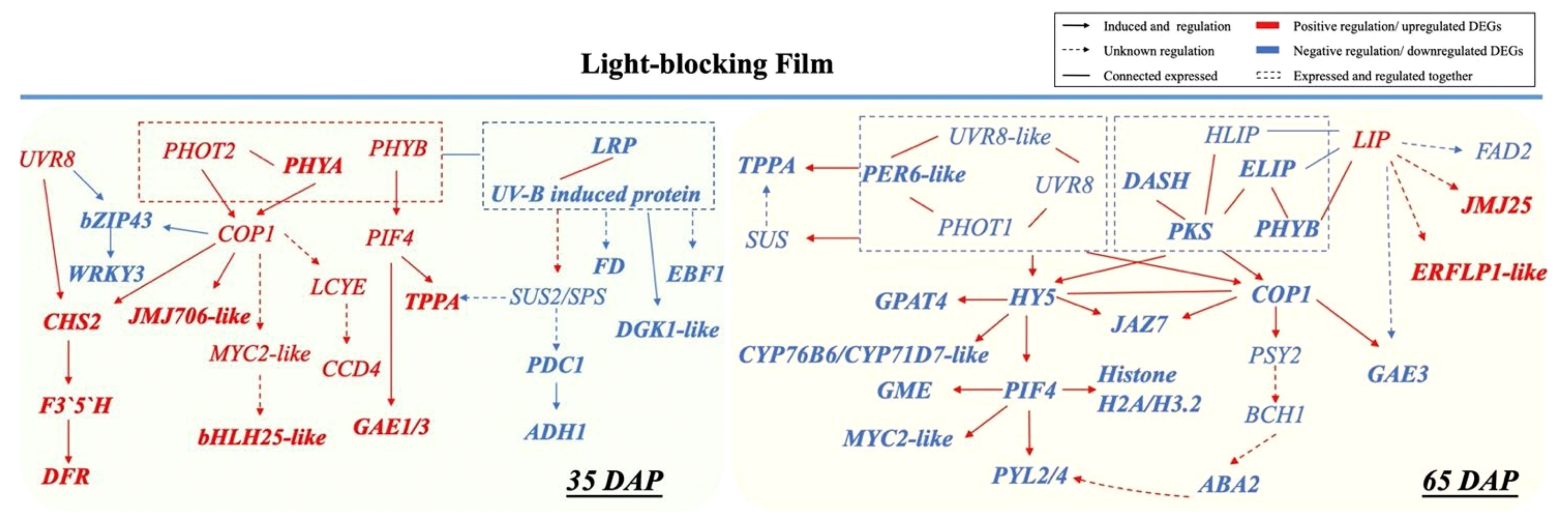
Cross-talk between light receptors regulated low-light adaptation in 35 DAP and 65 DAP of capsicum fruits under light-blocking film (LBF). *LRP*, *light-regulated protein*; *CHS*, *chalcone synthase*; *F3′5′H*, *flavonoid 3’*,*5’-hydroxylase*; *DFR*, *dihydroflavonol 4-reductase*; *JMJ706-like*, *lysine-specific demethylase*; *FD*, *ferredoxin*; *DGKA-like*, *diacylglycerol kinase A–like*; *EBF1-like*, *EIN3-binding F-box protein 1–like*; *SUS*, *sucrose synthase*; *SPS*, *sucrose-phosphate synthase*; *PDC1*, p*yruvate decarboxylase 1*; *ADH1*, *alcohol dehydrogenase 1*; *bZIP43*, *basic leucine zipper 43*; *WRKY3*, *WRKY transcription factor 3*; *MYC2-like*, *transcription factor myelocytomatosis 2–like*; *LCYE*, *lycopene epsilon cyclase*; *CCD4*, *carotenoid cleavage dioxygenase 4*; *PRE6-like*, *transcription factor PRE6–like*; *PYL2/4*, *abscisic acid receptor*; *DASH*, *Drosophila*, *Arabidopsis*, *Synechocystis*, *Human-type cryptochromes*; *PKS*, *protein PHYTOCHROME KINASE SUBSTRATE 1*; *HLIP*, *high-light–induced protein*; *ELIP*, *early-light–induced protein*; *LIP*, *light-induced protein*; *GME*, *GDP mannose-3*,*5-epimerase*; *PSY2*, *phytoene synthase 2*; *BCH1*, *beta-carotene hydroxylase 1*; *ABA2*, *xanthoxin dehydrogenase–like*; *GPAT4*, *glycerol-3-phosphate 2-O-acyltransferase 4*; *CYP71D7-like*, *cytochrome P450 71D7–like*; *CYP76B6*, *geraniol 8-hydroxylase–like*; *JAZ7*, *protein JAZ7*; *JMJ25-like*, *lysine-specific demethylase*; *FAD2*, *delta 12-acyl-lipid-desaturase–like*; *ERFLP1-like*, *ethylene-responsive proteinase inhibitor 1–like*; *GAE1/3*, *U 4-epimerase 1/3*.

A series of photoreceptors including PHYs, PHOTs CRYs, ZTLs, and UVR8 sense changes in light regulated by phytohormones (such as auxin and gibberellin) in response to shade conditions (Fraser et al., 2016; Cai et al., 2021; Sheng et al., 2022). Moreover, transcription factors PIFs have a dominant role as integrators of multiple light cues, driving fruit microclimate adaption (Kang et al., 2009; Luo et al., 2014; Pedmale et al., 2016). In initial fruit development, *PHYB* and PIFs regulate biosynthesis and translocation of auxin levels for rapid response to shade avoidance (Tao et al., 2008). We thus propose that PIF4 sensing the low light under LBF may corelated to fruit production in LBF adaptation via hormonal signaling auxin, ABA, and ethylene (**Figure 6**) (Rosado et al., 2016; Gramegna et al., 2019; Rosado et al., 2019). In this study, upregulated *PIF4* and *COP1* could respond to the low light signal perceived by *PHOT2* and *PHYA* with significant associations with upregulated *EXORDIUM-like* (*EXO*) for fruit development under low carbon availability [such as downregulated *SUS 2* (*SUS2*), *pyruvate decarboxylase 1* (*PCD1*), and *alcohol dehydrogenase 1* (*ADH1*)] (**Figure 7**) (Schröder et al., 2011; Vyse et al., 2022). *COP1* and *PIF4* can positively regulate the key genes in cell division and expansion including phytohormone (*JAJ706-like* and *MYC-like*), sucrose metabolism (*TPPA*) rather than storage (*PDC1* and *ADH1*), and antioxidants in fruit development (*CHS2* and *DFR*). Furthermore, the upregulated *PIF4* and *COP1* in 35 DAP could also modulate the low-light adaptation of capsicum plants by downregulating abiotic stress response genes, such as *hemoglobin-2* (*HB2*) and *serine/threonine-protein kinase* (*SAPK3-like*) under reduced sucrose transport and metabolism in LBF (**Figures 4**, **7**) (He et al., 2022; Chavan et al., 2023). Therefore, the orange capsicum variety has low-light tolerance via the upregulated *COP1* and *PIF4* and downstream genes to have strong impacts on fruit initial development (35 DAP) under LBF (Leng et al., 2013; Shinozaki et al., 2018; Razo-Mendivil et al., 2021).

**Figure 8:**
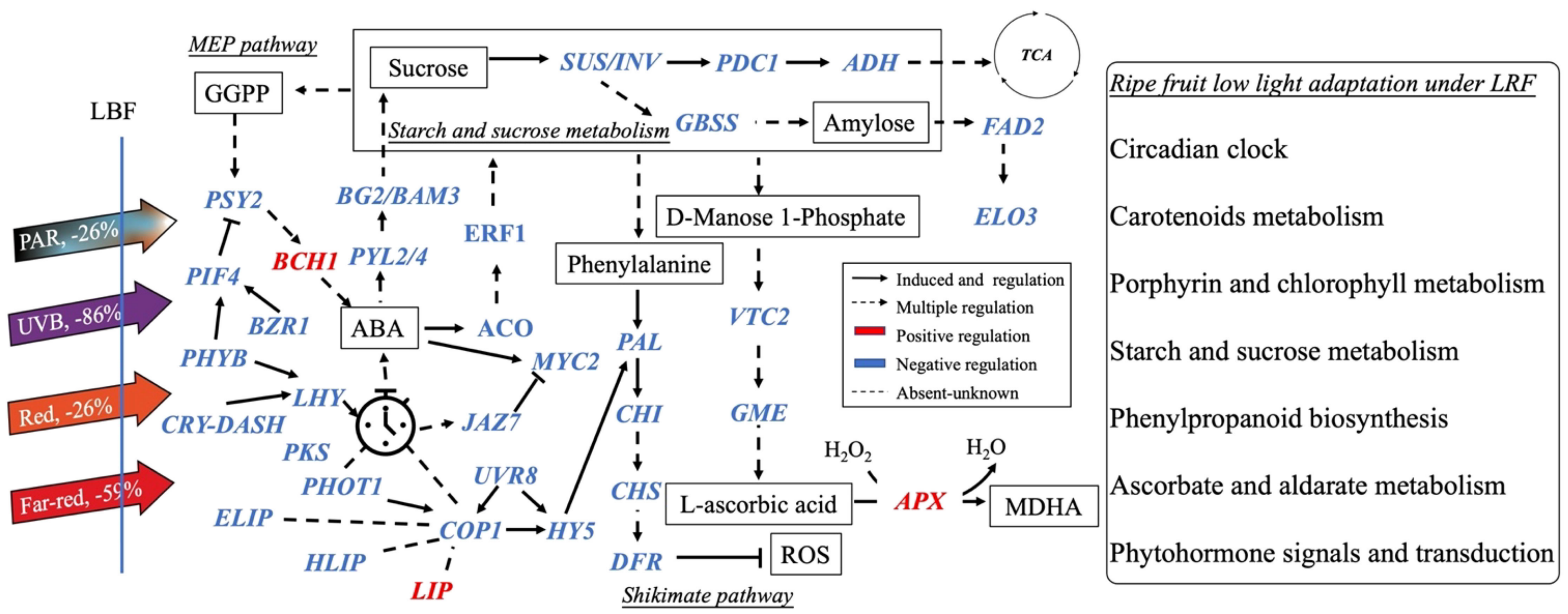
Schematic diagram of the main processes controlling the quality of ripening capsicum fruit (65 DAP) under light-blocking film (LBF). The dash lines mean the unknown regulation. Blue and red colors show the down- and up-regulated genes under LBF. *DASH*, *Drosophila*, *Arabidopsis*, *Synechocystis*, *Human-type cryptochromes*; *PKS*, *protein PHYTOCHROME KINASE SUBSTRATE 1*; *HLIP*, *high-light–induced protein*; *ELIP*, *early-light–induced protein*; *LIP*, *light-induced protein*; *PIF4*, *PHYTOCHROME-INTERACTING FACTOR 4*; *PAL*, *Phenylalanine ammonia lyase*; *CHS*, *chalcone synthase*; *CHI*, *chalcone isomerase*; *DFR*, *dihydroflavonol 4-reductase*; *SUS*, *sucrose synthase*; *PDC1*, *pyruvate decarboxylase 1*; *ADH1*, *alcohol dehydrogenase 1*; *BG2*, *glucan endo-1*,*3-beta-glucosidase 2*; *BAM3*, *beta-amylase 3*; *FAD2*, *delta 12-acyl-lipid-desaturase–like*; *ELO3*, *ELO HOMOLOG 3*; *MYC2*, *transcription factor myelocytomatosis 2*; *JAZ7*, *protein JAZ7*; *PYL2/4*, *abscisic acid receptor*; *BZR1*, *protein BRASSINAZOLE-RESISTANT 1–like*; *ERF1*, *ethylene-responsive transcription factor*; *ACO*, *1-Aminocyclopropane-1-Carboxylic Acid Oxidase*; *VTC2*, *L-galactose guanyltransferase*; *GME*, *GDP mannose-3*,*5-epimerase*; *APX*, *ascorbate peroxidase*; *PSY2*, *phytoene synthase 2*; *BCH1*, *beta-carotene hydroxylase 1*.

Casal (2013) and Yang et al. (2018) found that *PHYA* and *CRY1* participate in tomato shade avoidance. Here, we identified more light receptors and signaling factors, such as *ELIP*, *HLIP*, *PKS*, *LHY*, *LIP*, *CRY-DASH*, and *PER-like*, to be significantly responsive to LBF in 65-DAP fruit (**Figure 5B**, **Supplementary Table 11**). Under 65 DAP vs. 35 DAP, *ELIP* (in upregulated DEGs of control) and *HLIP* (in downregulated DEGs of LBF) can alleviate the oxidative stress in ripening process, which may signal the chloroplast-to-chromoplast transition in ripening fruit adaptation under low light generated by LBF (**Supplementary Tables 9**, **10**) (Müller et al., 2016; Timerbaev and Dolgov, 2019; Konert et al., 2022). *LHY* and *CRY-DASH* have functions in Circadian Clock–mediated changes in cell cycle regulation and chromatin organization (Facella et al., 2006; Müller et al., 2016). LBF-induced low light triggered the decline of light-regulated secondary metabolism in 65-DAP fruit under LBF (**Figure 8**, **Supplementary Table 12**). Except *COP1* and *PIF4*, low light triggers abiotic stress response to enhance *HY5* action, which was highly correlated to *COP1*, and may have feedback regulation with light receptors such as nuclear accumulation of *UVR8*, *CRY-DASH*, and *PHYB* (**Figure 7**) (Gangappa and Botto, 2014; Podolec and Ulm, 2018; Xu, 2020). Those downregulated light receptors and transduction signals were highly associated with the key genes in sucrose metabolism (*SUS* and *TPPA*), phytohormone transduction (*PSY2*, *ABA2*, and *PYL2/4*), which could affect the fruit ripening. Overall, light receptors participated in the adaptation of capsicum fruit to LBF, but it varies during fruit development. Although the functional basis of these photoreceptors and light-signaling components still requires future investigation, we propose that a combined effect of light receptors genes have an impact on the orange capsicum to LBF adaptation (**Figure 7**). Moreover, the expression of those light-signaling transduction DEGs was different between the developmental stages and significantly associated with plant hormone transduction and secondary metabolism pathway under LBF treatment.

### 4.2 Abscisic acid participates in the low-light adaptation specifically in ripe capsicum fruits

β-Branches of carotenoids serve as precursors for the synthesis of ABA, which is an important phytohormone regulating seed germination, plant growth, development, stress response, and non-climacteric fruit ripening (Osorio et al., 2012; De Wit et al., 2016; Feng et al., 2021). Here, we propose that downregulated light transcription factors *PIF4* and *HY5* under LBF could regulate the *PSY2* to control carotenoid synthesis in 65 DAP (Li et al., 2008; Casson et al., 2009). Furthermore, the majority content of zeaxanthin in capsicum fruit could be impacted by light changes via the regulation of *VDE* and *ZEP*, which continues to impact downstream ABA synthesis (Leng et al., 2013). For example, dark/light cycles affected ABA synthesis via ZEP, and the *VDE* expression was sensitive to the light environment (Thompson et al., 2000). However, *ZEP* and *VDE* were not found in the DEGs of both 35-DAP and 65-DAP fruits under LBF treatment (**Supplementary Table 5**). That could be the reason for no significant difference in total carotenoid content (He et al., 2022) and the percentage of zeaxanthin content in fruit at 65 DAP (**Figure 1F**) between LBF and control. The level of *NCED1/3* is an important regulatory step in stress-induced ABA synthesis (Li et al., 2011; Hou et al., 2018). Here, ABA synthesis genes, such as downregulated *ABA2* and *AO4*, rather than *NCED1/3*, had a significant relationship with light signal DEGs in 65 DAP after LBF treatment (**Figure 6**). Those reveal that ABA balances may also contribute to fruit adaptation in low light generated by LBF.

ABA homeostasis mediated by the ABA transporters and ABA signaling components is also important for fruit ripening (Hou et al., 2018; Li et al., 2021; Siebeneichler et al., 2022). Despite the direct molecular mechanisms of light receptors regulating ABA that are not currently resolved, many lines of evidence suggest that ABA plays a crucial role in the developmental and environmental adaptation processes of plants, such as upregulated *ABA* and *environment stress-inducible protein TAS14* and under LBF (**Supplementary Table 5**) (Chen et al., 2020). ABA catabolism (*CYP707A1*), reactivation (*BG genes*), and ABA signaling (*PYL2-like*, *PYL4*, and *ASR*) were significantly downregulated in 65 DAP under LBF, and these at the transcriptional level changes could affect active cellular ABA levels (**Supplementary Table 5**) (Li et al., 2013b). Furthermore, ABA signaling pathway significantly downregulated (**Figure 6**) together with light signals DEGs was examined in the same clusters (**Figures 2D**, **3E**), for example, downregulation of *PYL2-like* and *PYL4* significantly correlated to *COP1* and *PIF4* in 65-DAP fruits under LBF treatment (**Figure 7**). Thus, light-signaling transduction genes *COP1* and *PIF4* could be closely associated with ABA signal transduction for orange capsicum fruits ripening in LBF adaptation.

### 4.3 Low light trigged by LBF induces ripening relative compound trade-offs

Sucrose content is closely correlated with fruit growth and yield, which is usually affected by the light environment (Jia et al., 2013; He et al., 2022). As a source of fruit growth, the upregulated *SUS*, *beta-amylase 7* (*BAM7*) and *trehalose-phosphate* (*TPPA*) and downregulated *sucrose-phosphate synthase* (*SPS*; **Figures 7**, **8**, **Supplementary Figure 4**) in starch and sucrose metabolism of 35 DAP could be one of the reasons why there were no effects of LBF on the development and yield of orange capsicum fruit in our previous research (Chavan et al., 2023). During fruit development, starch and sucrose metabolism pathways participate in fruit adaptation when subjected to shade and covering materials (**Figure 8**) (Aroca-Delgado et al., 2019; Ji et al., 2020; He et al., 2022). This also coincides with those that are involved in starch and sucrose metabolism pathways than carbohydrate metabolism that is active in fruit under low-light conditions (**Supplementary Table 9**) (Li et al., 2013a; Chen et al., 2019a). For the 65-DAP fruit, the significantly downregulated genes in sucrose metabolism including *SUS*, *INV*, and *glucose-1-phosphate adenylyltransferase large subunit 1* (*AGPL1*) rather than sucrose transporters could impact the sucrose concentration (**Figure 8**, **Supplementary Figure 4**), such as significantly increased total soluble solids (TSSs; 4.4%; **Supplementary Table 13**) under low-light–induced LBF in our previous research (Watson et al., 2002; Chen et al., 2019a; He et al., 2022). That could be the differences in TSS composition, functional of DEGs in starch and sucrose metabolism, or subsidiary pathway, such as interactions of *PYL2/4* and *ERF1* regulated sucrose metabolism in fruit (Chen et al., 2019b; Baruah et al., 2021).

β-Branches of carotenoids are important pigments such as the high zeaxanthin accumulation for a diverse color of capsicum (**Figure 1**). The content of each carotenoid component in 65-DAP fruit and the colorimeter indexes (a* and b*) were significantly decreased and correlated with LBF in orange capsicum (He et al., 2022). That could be related to the LBF-induced downregulation of DEGs (including *FAD2* and *ELO3*) in fatty acid elongation and flavonoid synthesis gene (*CHS* and *CHI*) (**Figure 8**, **Supplementary Figure 2B**), which play an important role in the structure and fat solubility of carotenoids (Abbeddou et al., 2013; González-Ponce et al., 2018; Krauß et al., 2020). Furthermore, brassinosteroid (BR) biosynthesis participated in the orange fruit ripening under control (**Supplementary Figure 5**), which has been reported to be essential for growth and development and alleviate the detrimental effects of light stress on plants (Li et al., 2017; Jiroutova et al., 2018; Liu et al., 2020). Transgenic tomato lines overexpressing *Brassinazole Resistant 1* (*BZR1*) have increased transcript levels of *SlPSY1* and *SlZDS* and promoted the lycopene and carotenoids synthesis (**Figure 8**) (Vardhini and Rao, 2002; Liu et al., 2014). These indicate that low-light tolerance in the orange genotype may be associated with zeaxanthin accumulation.

ROS are also responsible for fruit ripening in non-climatic fruit as signaling molecules in coordination with phytohormones (Lytovchenko et al., 2011; Pérez-Ambrocio et al., 2018; Kim et al., 2021; Borbély et al., 2022). We found that light-induced ROS production promotes the expression of genes encoding antioxidant biosynthesis, such as *PAL*, *CHS*, *FLS*, *F3H*, *DFR*, and *GT*, maintaining plant growth and development (**Figure 8**). Low light under LBF affected phenylpropanoid biosynthesis and ascorbate and aldarate metabolism, which were evident by significantly downregulated DEGs such as *PAL*, *CHI*, *CHS*, *DFR*, and *GT* (**Figure 8**, **Supplementary Table 5**) (Akram et al., 2017; Pérez-Ambrocio et al., 2018). In the ascorbic acid biosynthesis pathway, GDP-D-mannose epimerase (GME) is a key enzyme in L-ascorbic acid biosynthesis concomitant with the fruit cell wall biosynthesis and softening in development (Alós et al., 2013; Gao et al., 2013). *GME*, *UGD*, and *UDP-glucuronate 4-epimerase 3* were significantly decreased in 65-DAP fruit, which could be associated with decreased ascorbic acid content (−14.1%) in orange ripening fruit in response to LBF (He et al., 2022). *L-ascorbate oxidase* (*AO*), *L-ascorbate peroxidase 6* (*APX6*), and *ascorbate transporter* (*AT*) contribute to the cellular redox homeostasis under low light (Yahia et al., 2001; Matteo et al., 2010; Haroldsen et al., 2011; Garchery et al., 2013; Siebeneichler et al., 2022). However, only *APX* and *AO* were found in DEGs of 65 DAP under LBF treatment, which indicated that low light could impact ascorbic acid synthesis in the orange cultivar.

## 5 Conclusion

We report that the differential expression of photoreceptor (specifically *PHOT2* and *PHYA*) and light-signaling genes (such as *COP1* and *PIF4*) could promote the low-light adaptation to regulate fruit development (35 DAP) under LBF (**Figure 7**). Moreover, the above light-signaling genes could mediate negative regulation of LBF on the circadian clock (*HLIP*, *ELIP*, and *CRY-DASH*) and plant hormone transduction (*NCED3*, *ABA2*, *AO4*, and *PYL2/4*), starch and sucrose metabolism (*SUS*, *SUC*, and *INV*), phenylpropanoid biosynthesis (*PAL* and *DFR*), and ascorbate and aldarate metabolism (*VTC2*, *AAO*, and *GME*) of 65-DAP fruit (**Figure 8**). The downregulated DEGs in phytohormone signaling pathways, phenylpropanoid biosynthesis, and aldarate metabolism could correlate with light signals and participate in ripe fruit LBF adaptation. However, a detailed understanding of how light regulates these photoreceptors and light-signaling components requires further study. We suggest that the changes in greenhouse light environment induced by the energy-saving LBF can have unexpected significant impact on a large number of genes that regulate capsicum fruit quality. Such information reveals the importance of a light environment for better-quality horticultural products under protected cropping, and the key light-signaling genes can guide plant breeders and growers to select and improve crop varieties that are more adapted to protected cropping conditions for sustainable and nutritious production under global climate change.

## Data availability statement

The datasets presented in this study can be found in online repositories. The names of the repository/repositories and accession number(s) can be found in the article/**Supplementary Material**.

## Author contributions

XH: Data curation, Formal Analysis, Investigation, Methodology, Software, Validation, Visualization, Writing – original draft. CS: Data curation, Investigation, Methodology, Validation, Writing – review & editing. CM: Investigation, Writing – review & editing. SC: Investigation, Writing – review & editing. YW: Data curation, Methodology, Visualization, Software, Writing – review & editing. WL: Investigation, Methodology, Writing – review & editing. NK: Investigation, Writing – review & editing. OG: Conceptualization, Funding acquisition, Supervision, Writing – review & editing. CC: Conceptualization, Funding acquisition, Supervision, Writing – review & editing. DT: Conceptualization, Funding acquisition, Project administration, Resources, Supervision, Writing – review & editing. Z-HC: Conceptualization, Formal Analysis, Funding acquisition, Project administration, Resources, Supervision, Writing – review & editing, Writing – original draft.
