# Supplementary material for: Novel transcriptome networks are associated with adaptation of capsicum fruit development to a light-blocking glasshouse film
Source: Front Plant Sci. 2023 Nov 6;14:1280314. doi: 10.3389/fpls.2023.1280314 (PMC10658010; doi:10.3389/fpls.2023.1280314)
Supplement: Supplementary file 1 [file DataSheet_1.docx]

Supplementary Figures


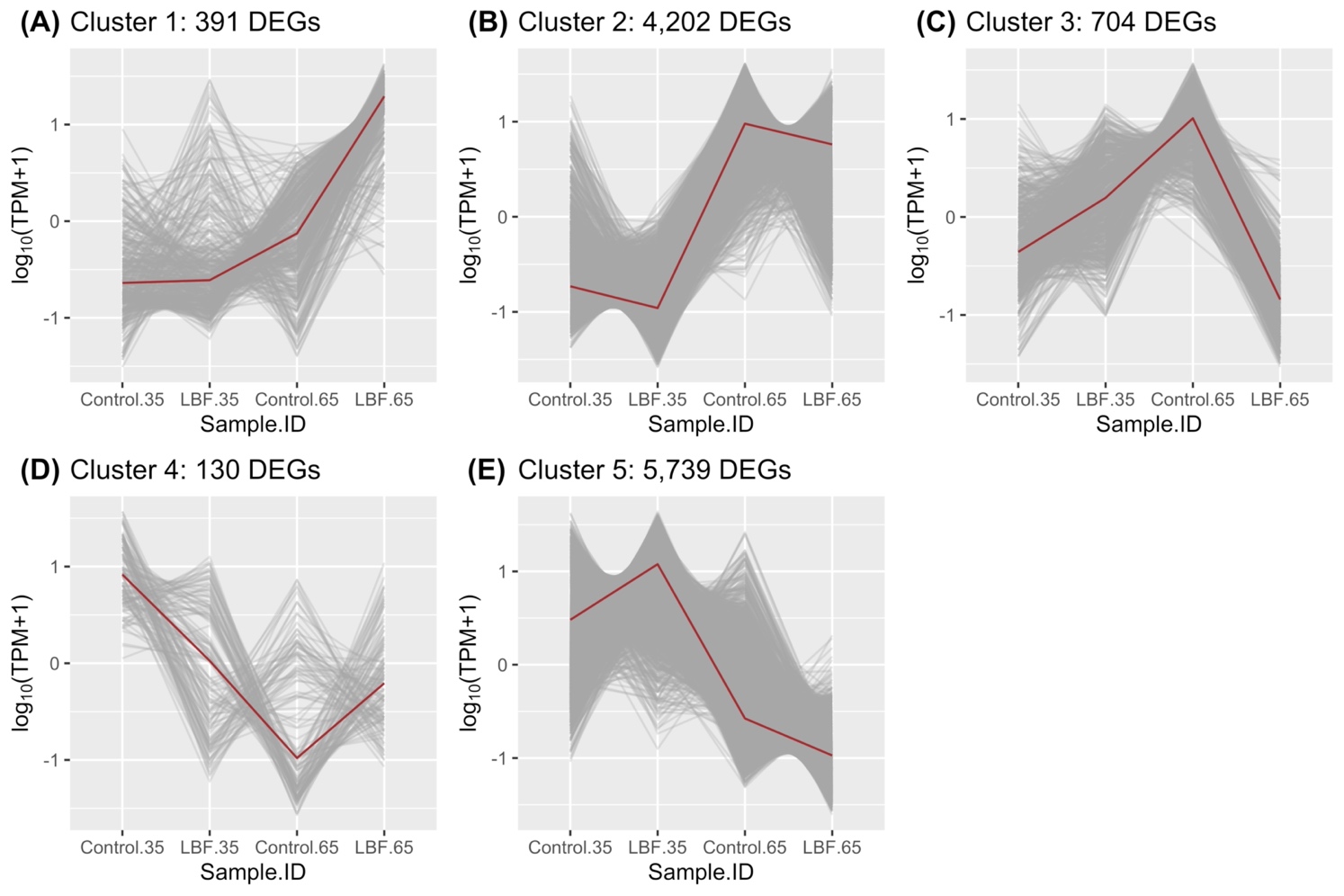


**Supplementary Figure 1.** Cluster analysis of DEGs heatmap. A K-means clustering analysis identified five clusters of DEGs according to their expression patterns. Mean expression values of each cluster are highlighted by the red line, and relative number of DEGs are labeled accordingly.


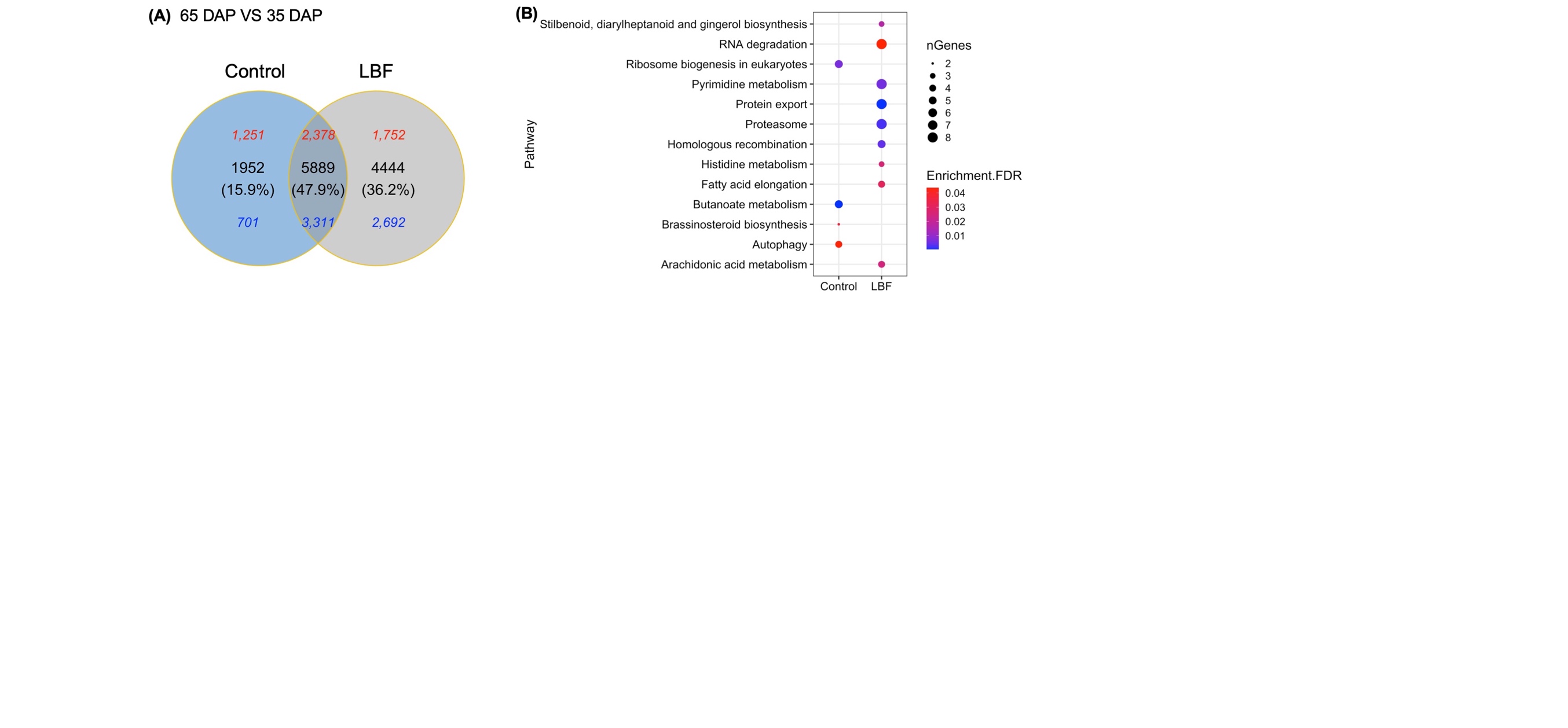


**Supplementary Figure 2.** Comparison of Capsicum fruit ripening process under 65 DAP vs 35 DAP under Control and Light-blocking Film (LBF). Venn diagrams (A) illustrates the DEGs under 65 DAP vs 35 DAP in Control and LBF. The number of up- and downregulated DEGs are labeled by red and blue, respectively. The bubble plot of Kyoto Encyclopedia of Genes and Genomes (KEGG) pathway analysis in Control and LBF under ripening process (65 DAP VS 35 DAP, B) presents the number of DEGs enriched in a particular pathway and relative color labeled by Enrichment FDR.


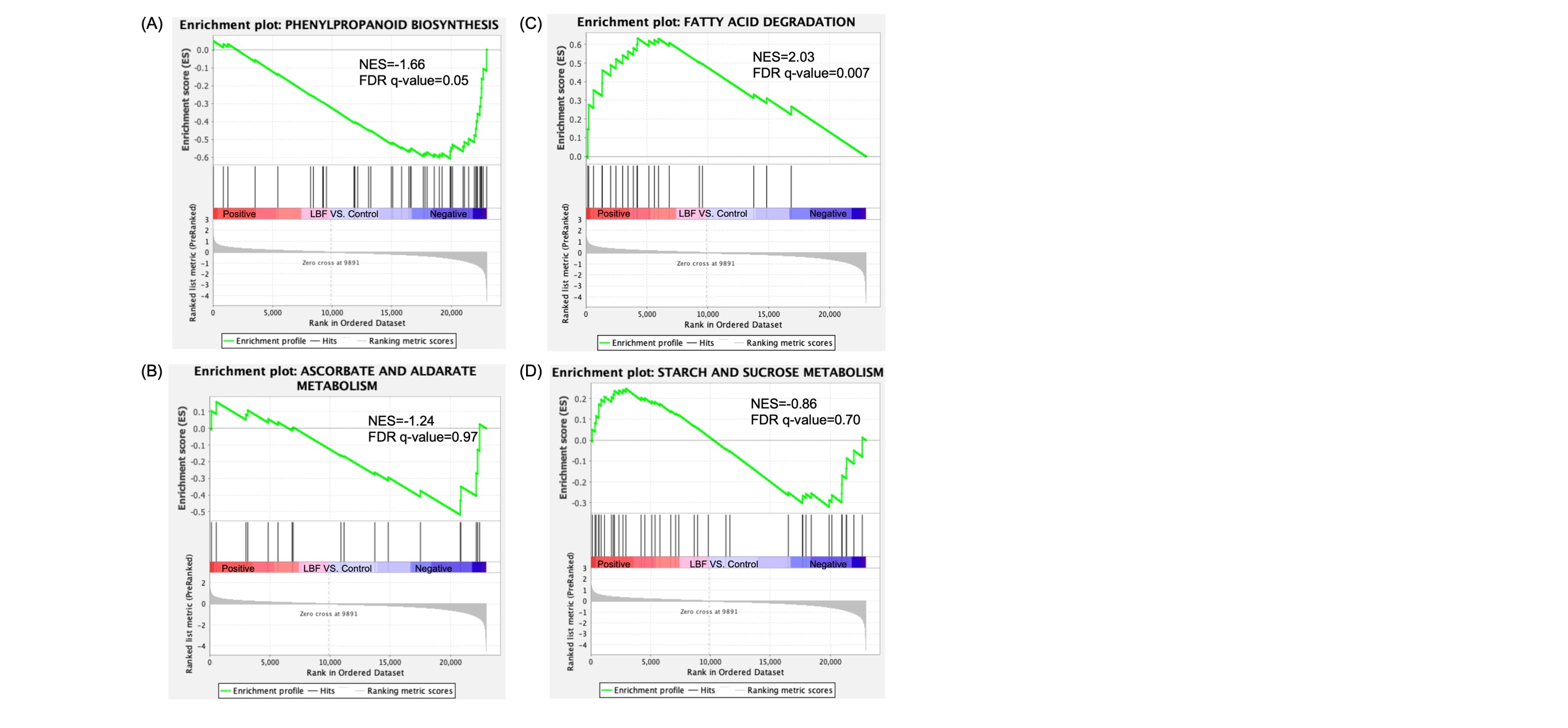


**Supplementary Figure 3.** Gene expression profile analysis by gene set enrichment analysis (GSEA). GSEA results of phenylpropanoid biosynthesis (A), ascorbate and aldarate metabolism (B), fatty acid degradation (C), starch and sucrose metabolism (D) in 65 DAP fruit under LBF vs Control. Positive (red) and negative (blue) ES indicate enrichment under LBF vs Control. Normalized enrichment scores (NES), p-value and false discovery rate (FDR) are indicated for each gene set. Y-axes indicate enrichment scores (top) and ranked list metric (bottom). X-axis bars represent individual genes of the indicated gene sets.


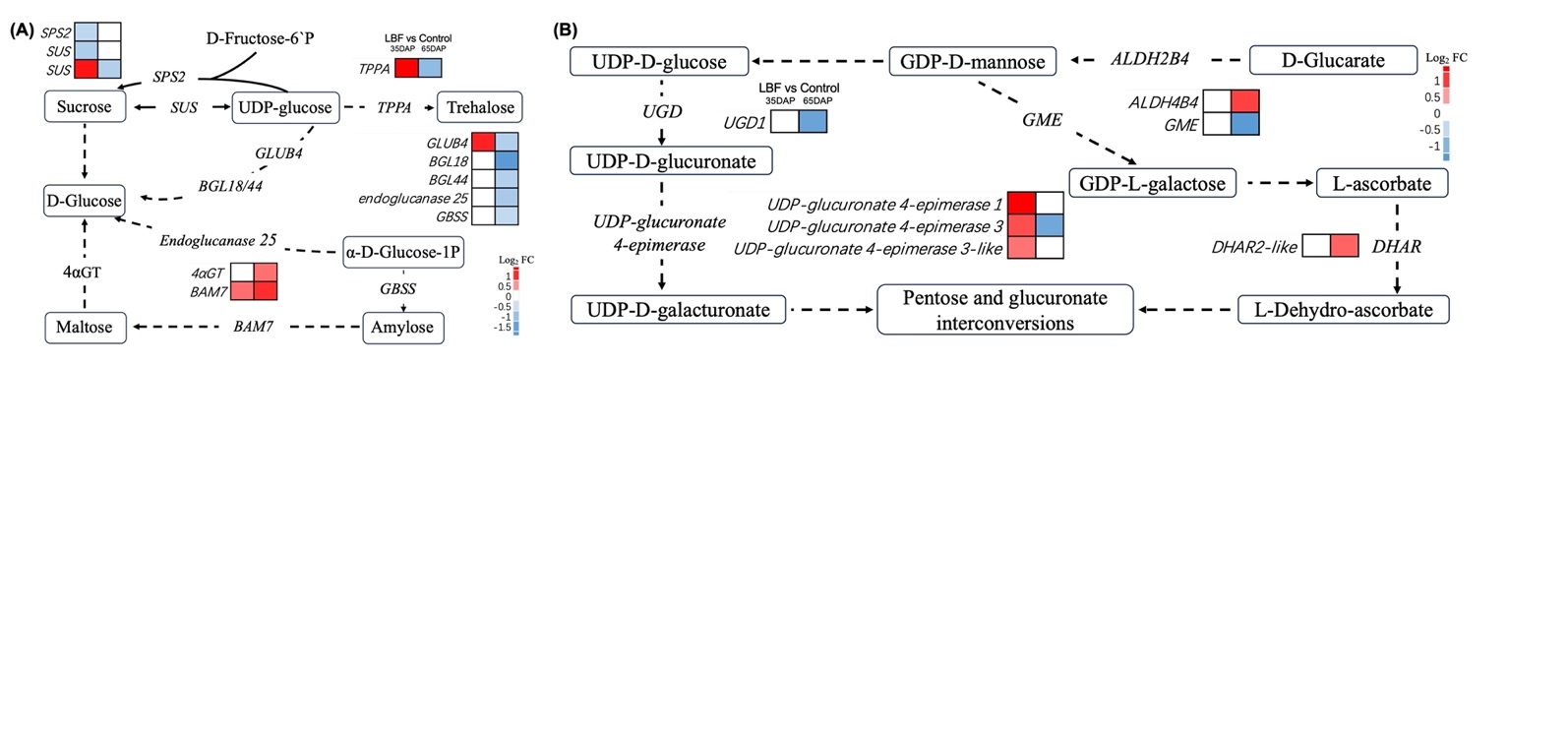


**Supplementary Figure 4.** Schematic network of starch and sucrose metabolism, ascorbate and aldarate metabolism in fruit under Light-blocking Film (LBF) vs Control. A diagram of starch and sucrose metabolism (A), ascorbate and aldarate metabolism (B) in 35 DAP and 65 DAP of Capsicum under LBF treatment. Positive (red) and negative (blue) ES indicate enrichment under LBF vs Control. Abbreviations: *beta-amylase 7* (*BAM7*), *glucan endo-1,3-beta-glucosidase 4-like* (*GLUB4*), *probable sucrose-phosphate synthase 2* (*SPS2*), *sucrose synthase* (*SUS*), *trehalose-phosphate* (*TPPA*), *4-alpha-glucanotransferase* (*4αGT*), *beta-glucosidase 18-like/444-like* (*BGL18/44*), *granule-bound starch synthase 2* (*GBSS*), *GDP mannose-3,5-epimerase* (*GME*), *aldehyde dehydrogenase family 2 member B4* (*ALDH2-B4*), *UDP-glucose dehydrogenase 1* (*UGD1*), *glutathione S-transferase* (*DHAR2-like*).
